# Evaluation of Digital Technologies Tailored to Support Young People’s Self-Management of Musculoskeletal Pain: Mixed Methods Study

**DOI:** 10.2196/18315

**Published:** 2020-06-05

**Authors:** Helen Slater, Jennifer N Stinson, Joanne E Jordan, Jason Chua, Ben Low, Chitra Lalloo, Quynh Pham, Joseph A Cafazzo, Andrew M Briggs

**Affiliations:** 1 School of Physiotherapy and Exercise Science Faculty of Health Sciences Curtin University Perth Australia; 2 Lawrence S Bloomberg Faculty of Nursing University of Toronto Toronto, ON Canada; 3 Child Health Evaluative Sciences, Research Institute The Hospital for Sick Children Toronto, ON Canada; 4 Institute of Health Policy, Management and Evaluation Dalla Lana School of Public Health University of Toronto Toronto, ON Canada; 5 HealthSense (Australia) Pty Ltd Melbourne Australia; 6 Centre for Musculoskeletal Outcomes Research Dunedin School of Medicine University of Otago Dunedin New Zealand; 7 Squawk Designs Perth Australia; 8 Centre for Global eHealth Innovation Techna Institute University Health Network Toronto, ON Canada; 9 Institute of Biomaterials and Biomedical Engineering Faculty of Applied Science and Engineering University of Toronto Toronto, ON Canada

**Keywords:** musculoskeletal pain, mHealth, eHealth, self-management, adolescent, mobile phone, smartphone

## Abstract

**Background:**

Digital technologies connect young people with health services and resources that support their self-care. The lack of accessible, reliable digital resources tailored to young people with persistent musculoskeletal pain is a significant gap in the health services in Australia. Recognizing the intense resourcing required to develop and implement effective electronic health (eHealth) interventions, the adaptation of extant, proven digital technologies may improve access to pain care with cost and time efficiencies.

**Objective:**

This study aimed to test the acceptability and need for adaptation of extant digital technologies, the pain*HEALTH* website and the *iCanCope with Pain* app, for use by young Australians with musculoskeletal pain.

**Methods:**

A 3-phased, mixed methods evaluation was undertaken from May 2019 to August 2019 in Australia. Young people aged 15 to 25 years with musculoskeletal pain for >3 months were recruited. Phases were sequential: (1) phase 1, participant testing (3 groups, each of n=5) of co-designed website prototypes compared with a control website (pain*HEALTH*), with user tasks mapped to eHealth quality and engagement criteria; (2) phase 2, participants’ week-long use of the *iCanCope with Pain* app with engagement data captured using a real-time analytic platform (daily check-ins for pain, interference, sleep, mood, physical activity, and energy levels; goal setting; and accessing resources); and (3) phase 3, semistructured interviews were conducted to gain insights into participants’ experiences of using these digital technologies.

**Results:**

Fifteen young people (12/15, 80% female; mean age 20.5 [SD 3.3] years; range 15-25 years) participated in all 3 phases. The phase 1 aggregated group data informed the recommendations used to guide 3 rapid cycles of prototype iteration. Adaptations included optimizing navigation, improving usability (functionality), and enhancing content to promote user engagement and acceptability. In phase 2, all participants checked in, with the highest frequency of full check-ins attributed to pain intensity (183/183, 100.0%), pain interference (175/183, 95.6%), and mood (152/183, 83.1%), respectively. Individual variability was evident for monitoring progress with the highest frequency of history views for pain intensity (51/183, 32.3%), followed by pain interference (24/183, 15.2%). For the goals set feature, 87% (13/15) of participants set a total of 42 goals covering 5 areas, most frequently for activity (35/42, 83%). For phase 3, metasynthesis of qualitative data highlighted that these digital tools were perceived as youth-focused and acceptable. A total of 4 metathemes emerged: (1) importance of user-centered design to leverage user engagement; (2) website design (features) promoting user acceptability and engagement; (3) app functionality supporting self-management; and (4) the role of wider promotion, health professional digital prescriptions, and strategies to ensure longer-term engagement.

**Conclusions:**

Leveraging extant digital tools, with appropriate user-informed adaptations, can help to build capacity tailored to support young people’s self-management of musculoskeletal pain.

## Introduction

### Background

Young people want access to health services that are tailored and responsive to their specific health needs, especially for those with chronic health conditions such as persistent musculoskeletal pain [1-3]. Digital technologies connect young people with health services and resources to support their self-care and promote positive health habits [4-6]. Furthermore, digital technologies are portable, customizable, and readily assimilate into young people’s daily routines [5].

This capability to connect is especially important for young people with chronic health conditions, including pain, during the critical transition from childhood to young adulthood [1,7]. Persistent pain can impose a significant and enduring health and economic burden on young people and their communities [8,9]. Australian data suggest persistent pain rates for young people approach those of adults (ie, approximately 20%) [8,10], whereas international data indicate higher rates for musculoskeletal pain (eg, 37% for back pain) [11-13]. Young people often fall through gaps during this developmental transition, with many failing to seek care and adopting unhelpful habits that increase the risk of ongoing health and social issues [6]. In Australia, this risk is compounded by young people exiting pediatric health services around the age of 16 years and failing to seamlessly integrate into adult health services, despite transitional frameworks [1,14,15]. Digital technologies may help to drive engagement, enabling timely access and broader reach to the *right* health care [1,14,16,17]. Currently, the lack of accessible, reliable digital resources tailored to the needs and preferences of young Australians with persistent musculoskeletal pain remains a health services gap [14,18]. Recognizing the intense resourcing required to develop and implement effective electronic health (eHealth) interventions, adaptation of proven digital technologies may be a more cost- and time-efficient approach [19].

To accelerate the implementation of digital technologies into the care of young Australians with musculoskeletal pain, we established a transnational partnership to leverage digital technologies already developed and tested for pain care. Both platforms were co-designed using best practice recommendations for eHealth design and implementation [20]. First, in Australia, we developed a digital resource, pain*HEALTH,* to support improved musculoskeletal pain care for adults [21-27]. Co-designed with consumers, using a policy-into-practice approach [28], aligned to contemporary musculoskeletal models of care and strategic health frameworks (the chronic conditions framework [29] and the national pain strategy [15]), pain*HEALTH* supports consumers accessing the right care, at the right time, by the right team. Impact evaluation demonstrated that consumers/caregivers and health professionals perceived pain*HEALTH* as supporting holistic self-management and cocare of musculoskeletal pain [30]. Second, in Canada, an integrated smartphone and web-based self-management program, *iCanCope with Pain,* was developed [16,31]. This digital platform addresses the self-management needs of young people with persistent pain by improving access to contemporary tailored pain education; providing practical strategies to manage pain, psychological well-being, and sleep hygiene; encouraging physical activity; and providing peer social support [3,16]. The platform is undergoing cultural adaptation and usability testing evaluation in Norway [32].

Trial evaluation in Canada is also underway to assess implementation success and effectiveness (such as pain intensity, pain-related activity limitations, and health-related quality of life) outcomes (clinical trial number: NCT02601755).

### Objective

Therefore, to avoid research waste and technology duplication, this study aimed to test the acceptability and need for adaptation of these two extant digital technologies for young Australians with musculoskeletal pain. The specific aims were as follows:

1. To test the acceptability and usability of a prototype derivative of pain*HEALTH* for young people with musculoskeletal pain
2. To test the acceptability and usability of the *iCanCope with Pain* app [16] for young people with musculoskeletal pain.

## Methods

### Study Design

**Figure 1:**
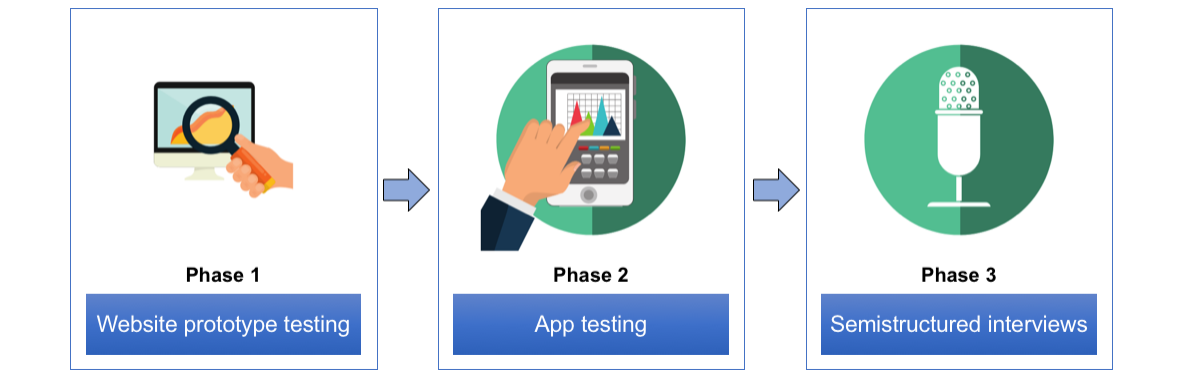
A 3-phased approach to the user testing was adopted, with all participants undertaking user testing of website prototypes (phase 1), pain app user testing (phase 2), and semistructured interviews on their experiences of using these digital technologies (phase 3).

This 3-phased, mixed methods evaluation was undertaken in Australia between May 2019 and August 2019. Phases were sequential, with phase 1 involving participant (user) testing of website prototypes (research aim 1). In phase 2, participants utilized the *iCanCope with Pain* app (hereafter *app*) over a 1-week engagement period (research aim 2). Finally, in phase 3, semistructured interviews were conducted to provide insights into the participants’ experiences of using these digital technologies (Figure 1). The study had institutional ethics approval, adhered to the Declaration of Helsinki, and aligned with reporting recommendations from the consolidated criteria for reporting qualitative studies: 32-item criteria (Multimedia Appendix 1) [33].

### Eligibility Criteria

Young people aged 15 to 25 years with musculoskeletal pain for >3 months and currently living in Australia were eligible to participate. Musculoskeletal pain included recurrent or persistent pain; however, pain at the time of enrollment was not a prerequisite for participation. These eligibility criteria were aligned to our previous research [1] and applied across all 3 study phases.

### Recruitment and Sampling

Participants were recruited from community sources, including arthritis consumer organizations, private health care practices, youth mental health services, via social media, electronic newsletters, flyers, and emails. Initial eligibility screening was undertaken using a web-based survey platform Qualtrics. The survey instrument was informed by our previous research on young people with musculoskeletal pain [1,16], with minor iterations, and was piloted by members of the research team (HS, AMB, J Chua, and JNS) before deployment to the field. No researcher had an existing relationship with any participant.

### Consent

Young people meeting all 3 inclusion criteria were requested to provide consent directly through the Qualtrics web platform. Participants aged 15 to 17 years were advised to discuss consent with their guardian/parent before consenting to participate and were also invited to have a guardian/parent present for any, or all, phases of the study.

### Protocol

The participants meeting the inclusion criteria and providing consent completed an initial web-based survey to capture demographics and clinical characteristics, including area(s) of their pain, pain duration, and pain diagnosis. The Örebro Musculoskeletal Pain Screening Questionnaire-Short Form (ÖMPSQ-SF) [34] was included as a measure of pain-related disability. The ÖMPSQ-SF has been validated for use in primary care [35] and used for previous clinical pain research in this age group [1]. Items are scored 0 to 10; 0 refers to the absence of impairment and 10 to severe impairment. A total of 3 items are reversed in order for all the questions to be oriented in the same direction. Total scores range between 1 and 100, with a score >50 indicating higher estimated risk for future (work) disability. Given the age range for our cohort, we modified the wording for items where *work* was mentioned to include *work/study*. Once participants completed the web-based survey, they were contacted to schedule a time for phase 1 of the user testing.

Participants were allocated sequentially to 3 testing groups (each comprising 5 participants). Groups were run in series, with group allocations remaining consistent across all 3 phases of testing (as all participants consented to complete all 3 phases). The purpose of serial testing in phase 1 was to enable iterative cycles of formative feedback used to guide prototype refinements before the subsequent testing group, an approach consistent with best practice recommendations for digital user testing [16,20]. The period of testing across all 3 phases for a group ranged from 11 to 26 days. At the completion of phase 3, all participants received an AUD $100 (US $65) gift voucher.

#### Phase 1. pain*HEALTH* and Prototype Website User Testing

For this phase of user testing, the existing pain*HEALTH* platform was used as a *control* website. This allowed users to evaluate 2 newly developed website prototypes against a fully functional comparator. As pain*HEALTH* was co-designed with adults, rather than younger Australians, we did not expect participants to easily relate to this website, but rather to consider features and content that might be helpful on a youth-focused platform. The prototypes were informed from our previous research using insights from young people with musculoskeletal pain about their needs and preferences for digital tools [1]. The specific tasks are outlined later in the paper.

We implemented *Lookback* as the test platform, using the *LookBack Live* option. *LookBack Live* allows users and researchers to engage and communicate together in real time with a website (in this case, pain*HEALTH* and website prototypes) via a shared screen, with reactions, comments, behaviors, and interactions captured via downloadable audiovisual recording. This platform provided a good *fit for purpose*, enabling flexible scheduling, remote user access, and participants use of their own computers or mobile devices without the need for additional cables or cameras. User insights are denoted by a researcher’s use of *time stamps*, with additional capacity for taking technical notes within the test platform that can later be extracted in text and recorded form. A rapid cycle of prototype iteration was undertaken at each group’s completion of user testing.

Before implementing the user testing, the LookBack Live platform and user tasks were piloted by members of the research team (HS, AMB, JNS, and CL). Minor amendments were made to the user tasks to improve clarity regarding tasks. Participants were scheduled to a time slot, emailed a unique LookBack Live invitation link, and provided with instructions for the session. Participants were informed that the user testing was about the prototypes (and not themselves) and they “can’t do or say anything wrong” and to “think aloud” as much as possible. The total mean duration of the web-based prototype testing was between 60 and 90 min.

**Figure 2:**
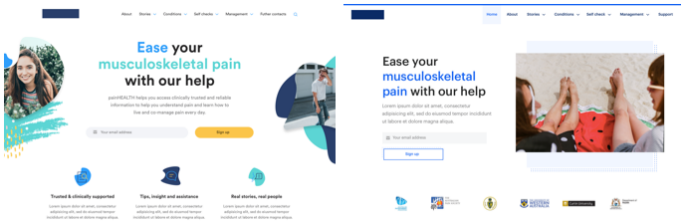
Screenshots of the 2 prototype websites: to the left, the round, soft version is shown, and to the right, the geometric version.

Participants were tasked with evaluation in the following order:

- The pain*HEALTH* website: This website was used as a control site to help orient participants toward the look and feel, functionality, and content of a contemporary, consumer-focused, Australian musculoskeletal pain website [30]. Users were guided through 4 tasks covering interactive content (pain conditions and stories, self-checks, and pain management) on musculoskeletal pain care.
- Two newly developed prototype websites (Figure 2): Prototype 1 was characterized by soft round shapes with a palette of purple/yellow/pink, and prototype 2 featured a geometric design with a blue/black palette). Content for the prototypes was adapted from the pain*HEALTH* website for the evaluation tasks. The prototype websites were powered by InVision (digital product design).

Participants completed the same 4 tasks across each of the 3 websites (Multimedia Appendix 2). Each test task was contextualized by: (1) a background statement about the nature of the task, (2) instructions to guide the specific task required, (3) specific questions about each task to gauge feedback, and (4) any additional feedback. The 4 test tasks were as follows

1. Find and watch *Daniel’s pain story*: This video/text content captured a young person’s narrative around their experiences with low back pain.
2. Find and complete the ‘*Örebro Musculoskeletal Pain Self-Check*’: This self-check questionnaire is designed for use across musculoskeletal pain conditions to predict the risk of higher estimated disability [34], and users can print out their score to share with health professionals.
3. Find and read the *Making Sense of Pain* management module: This content is focused on reasons why pain can persist beyond normal tissue healing times and the need for tailored holistic and integrated pain care.
4. Find *Further assistance*: This page provides a list of West Australian pain services and links to a cross-discipline health professional listing managed by the Australian Pain Society.

These test tasks were designed to reflect user-centered eHealth design principles [20] and criteria that predict real-world user engagement [20,36,37], specifically:

- Navigation
- Usability (functionality and ease of utilization)
- User engagement (content presentation, interactive, not irritating, targeted/tailored/personalized, captivating, and relatability)
- Content (appropriate level of literacy, credible, clear, and concise)
- Acceptability (design look and feel, meets the expectations of young people with musculoskeletal pain, motivating, and likability).

Engagement criteria were intentionally mapped across the task tests, with some overlapping of criteria (Table 1) to enable meaningful interpretation of outcomes and inform prototype iteration.

**Table 1:** Four test tasks were mapped, as per ticks, to reflect specific electronic health evaluation criteria.

Design and quality criteria	Test tasks			
	Daniel’s story	Self-check^a^	Making sense of pain	Further support
Navigation	✓	N/A^b^	N/A	✓
Usability	N/A	✓	N/A	✓
User engagement	✓	✓	✓	✓
Content	✓	✓	✓	✓
Acceptability	✓	✓	✓	✓

^a^Self-check refers to validated self-report questionnaires.

^b^N/A: not applicable.

#### Phase 2. *iCanCope With Pain* App User Testing

For phase 2, we adapted the framework developed by Stinson et al [16] for guiding young people’s use of the *iCanCope with Pain* integrated web- and smartphone-based app [16,31]. The specific app features [16] tested included:

- Symptom(s) check-in
- Structured goal setting to improve pain and function
- An interactive toolbox for pain coping strategies
- Access to papers to support users understand and manage their pain, mood, sleep, activity, and social function.

Participants were contacted by email within 2 days of completion of phase 1 and provided with the following standardized information (Multimedia Appendix 3):

- An overview of the *iCanCope with Pain* app (why it was developed, how it is currently being used, and by whom) and a link to the *iCanCope with Pain* website
- Features of the app used to support the self-management of pain (tracking and monitoring function; tracking your sleep, mood, physical activity, and energy levels; setting realistic goals; coping tools; and resources to support care)
- Instructions on how to access the app (via the app store) and sign in using their unique independently generated user name and password.

Instructions also outlined 4 key tasks for participants to complete:

1. Setting up their individual user profile (pain areas, triggers, pain intensity, interference, mood, sleep, physical activity, and energy)
2. Setting up their individualized goals aimed at improving their functioning in 5 main areas (physical activity, sleep, social, mood, and energy) and monitoring their progress against these goals
3. Symptom tracking over the 7 days to self-monitor progress (including pain, interference, mood, sleep, physical activity, and energy) and captured in the form of daily *check-in* reports
4. Accessing and exploring the library and the community resources designed to support their pain management (topics including nutrition, mood, sleep, work and study, social activities, self-worth, coping with pain, dealing with setbacks, talking to friends, employers, and teachers about pain).

Once set up and having completed these initial tasks, participants were encouraged to use the app daily to self-track and monitor their progress over the following 7-day period, while also using the app as they wished. Data extraction was performed by a team member (QP) blinded to phase 1 outcomes.

#### Phase 3: Semistructured Interviews

In the final phase, individual in-depth interviews were undertaken with participants about their experiences of using these 2 digital tools (website and app). The interview schedule was developed by a multidisciplinary research team (including clinical researchers with experience of musculoskeletal pain [HS, JNS, AMB]) and informed by prior user testing of these digital platforms [16,30] and our research on young people experiencing musculoskeletal pain [1,14] (Multimedia Appendix 4). Before the interview, participants were contacted (JEJ) to outline the interview process and schedule a time for the interview, in a quiet location chosen by the participant. All 15 interviews were conducted via teleconferencing by a senior qualitative researcher (JEJ) with experience in interviewing young people with musculoskeletal pain and who was independent of phase 1 and 2. No participant requested a support person during the interview. Interviews were audio recorded (duration ranged between 14 and 36 min, mean duration 21 min) and transcribed verbatim. Verbatim transcripts were checked for accuracy.

### Data Analysis

#### Phase 1

User insights and feedback were extracted at the completion of each group’s cycle of testing. These text-based group data were recorded in an Excel spreadsheet, aggregated (BL), and mapped against the established eHealth criteria (HS; Table 1). These data were discussed by members of the team (HS and BL), and a consensus reached about what iterations should be implemented. Rapid cycles of prototype iteration were then undertaken and implemented before the next cycle of group testing. Therefore, 3 cycles of prototype iteration were undertaken and implemented (between groups 1 and 2, between groups 2 and 3, and 1 final cycle at the completion of group 3 testing).

#### Phase 2

Participant engagement over the 7 days of testing was captured and evaluated through an Analytics Platform to Evaluate Effective Engagement (APEEE) with digital health interventions [38]. APEEE is a dynamic, real-time analytic platform that captures and characterizes user app engagement. Outcomes from APEEE included the following:

- Total number of full check-ins (completion of all relevant domains), time taken to check-in (min/second), check-in by domain (pain activity, mood, physical activity, sleep, and energy). Note that sleep can only be logged once per day.
- History views (number of times each symptom domain was reviewed: pain, interference, activity, mood, sleep, and energy). Note that pain intensity is the default history domain, with users having to toggle to view other domains.
- Goals set (number and domain: activity, sleep, energy, mood, social).
- Library articles accessed (number and type).

#### Phase 3

The 15 verbatim transcripts were analyzed (coded) by 1 researcher (JEJ) in 3 sequential stages (ie, 3 sets of 5 transcripts) using inductive and deductive approaches. In the first stage, 5 transcripts were analyzed using a general inductive analytical approach, where codes were directly derived from the data or from *the ground up*, without starting from a prior theoretical understanding of the issue being explored. For the second stage, the coding framework inductively derived from the first five 5 transcripts was then utilized to deductively code the second set of 5 transcripts. When a new topic emerged from the data, a corresponding code was developed inductively. In the third stage, to verify data redundancy, the revised coding framework was applied to the final set of 5 transcripts. No new codes were added to the framework in this final stage of the analysis. By utilizing a combined inductive and deductive analytical approach, which involved a constant comparison of data over a period until no new topics emerged (data redundancy), a comprehensive profile of participant preferences and experiences was developed. Following the finalization of the coding framework, codes were reviewed, and key themes and corresponding subthemes were developed through an iterative process of grouping codes into concepts, reviewing transcripts, and refining themes. The interview schedule was intentionally divided into 4 parts: (1) general background/context (questions 1 and 2); (2) perceptions of the pain*HEALTH* website functionality (question 3); (3) perceptions of the *iCanCope* *with Pain* app functionality (question 4); and (4) perceptions related to the acceptability, use, and implementation of the 2 digital tools generally (questions 5-13). This enabled the analysis of transcripts to be undertaken for each part of the schedule as mutually exclusive categories. Key themes and subthemes aligning to each part of the interview schedule were then reviewed (HS, AMB, and JEJ) and grouped under overarching (meta) themes [39]. One-third of the interview transcripts (n=5) were independently analyzed (JNS and CL) to confirm themes identified, and where necessary, refined to reach consensus, and confirm the construct validity. Data were structurally organized to present themes in a logical explanatory scheme [40].

We evaluated the extent to which our inductively derived data mapped to an evidence-based eHealth roadmap (the latter, extensively reviewed elsewhere) [20]. The roadmap takes a holistic approach to the development of eHealth technologies, integrating persuasive health technology theories with business modeling (efficient, effective, and sustainable) to improve the uptake and impact of eHealth technologies in practice. The roadmap is agnostic to digital platforms, is inherently fluid, interactive, and allows for a cyclical approach to the co-design (acceptability, functionality, and usability) and implementation of digital technologies in real-world settings. The roadmap provided a good *fit for purpose*, the plurality allowing for analysis of content relevant across both digital platforms (specifically, contextual inquiry, user requirements, value specification, co-design, and operationalization, referred to here as implementation).

## Results

### Demographic and Clinical Characteristics

A total of 20 potential participants met the inclusion criteria and consented to participate (Table 2). Of these 20, 15 (75%) participated and completed all 3 phases of the study, and 5 recruits did not start the study for various reasons (2 unwell, 2 for personal reasons, and 1 did not respond to email contacts or phone follow-up). The majority of these 15 participants were female (12/15, 80%) and resided in Western Australia (13/15, 87%). The mean age of these participants was 20.5 (SD 3.3) years.

For those participants with a confirmed medical diagnosis (10/15, 67%), musculoskeletal conditions included Ehlers-Danlos Syndrome (n=2), fibromyalgia (n=4), endometriosis (n=1; comorbid with back pain), rheumatoid arthritis (n=1), scoliosis (n=1), sacroiliitis (n=1), hip labral tear (n=1), and low back pain with nerve-related leg pain (n=1). For those reporting no confirmed diagnosis, conditions primarily related to low back/neck pain or upper/lower limb muscle or joint pain.

**Table 2:** Demographic and clinical pain characteristics of consenting participants (N=20).

Demographic/clinical pain characteristics			Consenting and participating (n=15)	Consenting and not participating (n=5)
**Age (years)**				
	Mean (SD)		20.5 (3.3)	22.6 (3.1)
	Range		15-25	18-25
Gender (female), n (%)			12 (80)	4 (80)
Urban/rural, n (%)			13 (86)	1 (20)
English as a first language, n (%)			14 (93)	5 (100)
**Highest current level of education completed, n (%)**				
	University		4 (26)	1 (20)
^ ^	TAFE^a^		1 (6)	2 (40)
	Year 12 (tertiary entrance)^b^		6 (40)	1 (20)
	Year 12 (other)		2 (13)	1 (20)
	Less than 3 year secondary		2 (13)	0 (0)
**Currently at, n (%)**				
	School		4 (26)	0 (0)
	University or TAFE		8 (53)	1 (20)
	Unemployed		0 (0)	2 (40)
	Employed (volunteer or paid work)		3 (20)	2 (40)
**Pain**				
	Diagnosis from health professional (yes), n (%)		10 (66)	4 (80)
	**Duration of pain (years)**			
		Mean (SD)	6 (6)	8 (8)
		Range	0.3-22	2.5-18
	**ÖMPSQ-SF^c^**			
		Mean (SD)	47 (14)	62 (6)
		Range	27-74	52-66
	**Area(s) of pain^d^, n (%)**			
		Neck pain	6 (40)	3 (60)
		Mid back	7 (46)	3 (60)
		Low back	8 (53)	4 (80)
		Hips	5 (33)	2 (40)
		Knees	3 (20)	2 (40)
		Ankles	4 (26)	0 (0)
		Shoulders	4 (26)	1 (20)
		Elbows	1 (6)	1 (20)
		Wrists/hands	11 (73)	1 (20)
		All over pain (muscles and joints)	6 (40)	2 (40)
		Other pain^e^	10 (66)	3 (60)

^a^TAFE: Technical and Further Education Institutions.

^b^Pathway for university entrance.

^c^ÖMPSQ-SF: Örebro Musculoskeletal Pain Screening Questionnaire-Short Form, possible score 1 to 100.

^d^Total count may be greater than the number of participants as more than one area of pain could be nominated.

^e^Areas of pain nominated in free text included abdominal pain (n=3), coccygeal pain (n=1), migraine (n=3), gastrointestinal issues (n=2), dysmenorrhea (n=1), and nerve pain (n=1).

### Phase 1: pain*HEALTH* and Prototype Website User Testing Outcomes

Outcomes from user testing of websites provided rich insights across the 3 groups. Outcomes are summarized sequentially by groups (1, 2, and 3) with recommendations derived to inform each cycle of prototype iteration, shown in Textboxes 1 to 3, respectively. A comprehensive tabulation of these data mapped by group to design criteria (navigation, usability, user engagement, content, and acceptability) with supporting user insights and quotes is provided in Multimedia Appendix 5.

For group 1, outcomes are summarized in Textbox 1. Participants leaned more strongly toward preferring the *round and bright* prototype variation. Compared with the control site, participants liked the use of dropdowns (easier navigation) and self-check functionality enhancements (better user experience for quiz completion). The *Further Support* menu did not resonate well with participant expectations, as this suggested *technical support* for some. Simplifying language and the use of a glossary were perceived as enhancements that would improve user engagement. On the basis of these outcomes and discussions with members of the team (AMB, JNS, BL, and HS), a consensus decision was reached to proceed with the *round and bright* prototype only. Collectively, these data informed the first rapid cycle of prototype iteration, implemented before group 2 testing.

**Recommendations for prototype iteration based on insights from group 1 participants.**

- Navigation: add drop-down menus throughout the prototype website to facilitate navigation and improve user engagement.
- Usability: *self-check* improvements included providing an explanation about what the self-check results mean to help reduce anxiety and fear about the results and the addition of explanatory text about who to see (health professional) and why.
- Content and user engagement: simplifying text, chunking text, and use of bold quotes to highlight key messages.
- Acceptability: *Further Assistance* changed to *Further Help* to capture both the listing of resources, services, and links to health professionals.

Group 2 insights indicated overall positive responses to the colorful prototype iterations and informed further recommendations for iteration (Textbox 2; Multimedia Appendix 5). Participant insights highlighted the ease of navigation, usability (functionality), engagement, content, and acceptability compared with the pain*HEALTH* website. Recommendations to inform the second cycle of prototype iteration were implemented before group 3 testing.

**Recommendations for prototype iteration based on insights from group 2 participants.**

- Navigation: enhance the navigation bar to ensure it is *sticky* on every page (ie, the navigation bar follows the user as they move up and down a page); implement hyperlinks to open external websites in a new tab; improve identification of external hyperlinks versus internal; add global navigation to the self-check, with the removal of progression steps; inclusion of a progress bar/percentage; remove lifestyle image on self-check start page; and use a smaller icon to reduce the need for users to scroll to start the self-check quiz.
- Usability: for the self-check, revise the text to better explain what the self-check is, why a user would complete it, and how they can use this information.
- User engagement: introduce categories within the management modules to better direct users to relevant content and further optimize and enhance user engagement and acceptability.
- Content: review website content and optimize for a younger reading audience (the appropriate level of health literacy) with even more *chunked content* and amend *Further Contacts* further, adding 3 tabs to be meet user expectation about related page content and to reduce the volume of contacts listed on the page into clear groupings to be easily scannable.
- Acceptability: color palette optimization to improve the relatability for the targeted age range using a softer palette of welcoming blues and engaging yellows that still *feels* young; drop the pinks and purples to ensure accessibility for color-blind individuals.

Group 3 participants indicated a positive overall response to the prototype compared with the control site (Textbox 3; Multimedia Appendix 5), with all indicating that the color palette was engaging and fun and nicely targeted toward a younger demographic. Feedback highlighted an improved user experience in navigation, with no further issues reported. Self-check functionality enhancements were well received. One participant raised the possibility of implementing screen reader functionality to help improve readability (when required). Compared with the control site, the use of chunked information and larger font size made for easier readability. Compared with the control website, this prototype was perceived by group 3 participants as providing a good example of a holistic, integrated approach to young people’s pain, making it easy to find, research and understand their pain. To further support and explain content, the use of more illustrations/videos within the management modules was proposed. Overall, participants found the website acceptable, reporting the look and feel as engaging, fun, relatable, and appropriate to their demographic. Collectively, group 3 insights guided the final prototype iteration recommendations.

**Final recommendations for prototype iteration based on insights from group 3 participants.**

- Usability: consider screen reader functionality to support users or consider extracting the audio from website videos, loading these as audio files. For longer form content, capturing readings of content, to then create downloadable audio files.
- Content: the use of a sidebar section that can act as an *anchor link* that is sticky and follows the user up/down the screen and scrolls their viewpoint to the contact area to further assist finding relevant contacts.
- User engagement: embed images that are resonant of the specific pain management module the content is about (eg, an image or icon of an activity or walking for *Movement with Pain*) to help differentiate the management module content and better assist, engage, motivate, and relate to the user.

### Phase 2 Outcomes: *iCanCope with Pain* App

**Figure 3:**
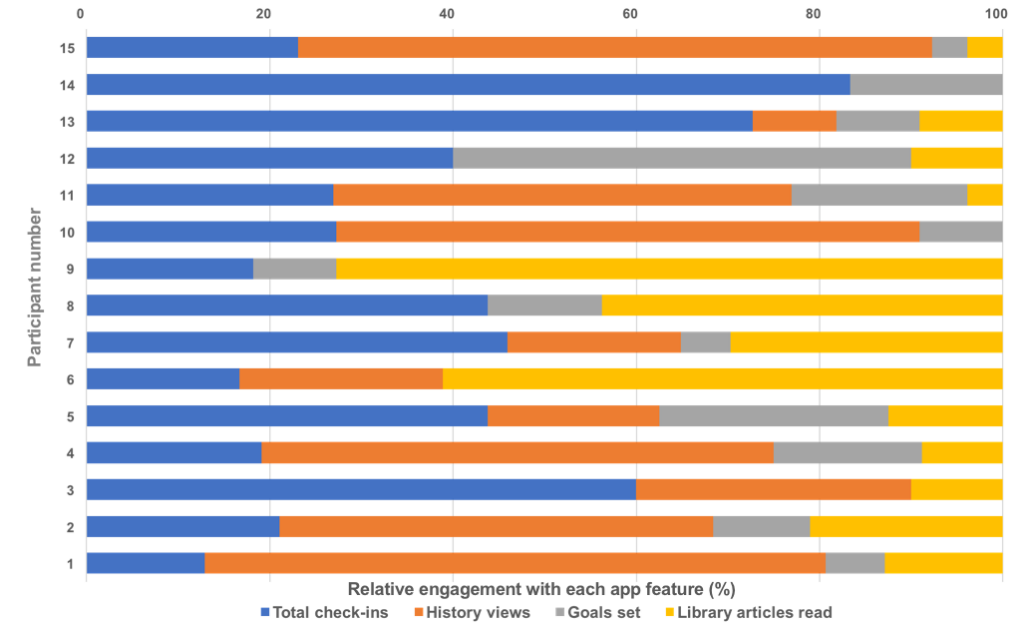
Individual-level data are shown for participants 1 to 15 (vertical axis). Relative user engagement (horizontal axis, proportional frequencies, and ranges for each domain) is presented across 4 key domains: (1) total (full) check-ins (blue), range 2-17; (2) history views (bright orange), range 0-38; (3) goals set (gray), 0-11; and (4) library articles accessed (light orange), range 0-11. Note that it is the variable relative engagement of each individual with the features of the app.

Participants’ engagement data for the use of the *iCanCope with Pain* app are summarized in Table 3 and Figure 3. The group mean (SD) and median frequencies for specific app features were: check-ins, 7.3 (4.0), 7.0; history views, 10.5 (12.8), 4.0; goals set, 2.8 (2.8), 2.0; and articles accessed, 4.2 (3.9), 2.0. Over the week of app use, all participants checked in, with 183 check-ins initiated. Of these, 59.6% (109/183) were full check-ins, meaning completion of all 5 domains (sleep, mood, physical activity, and energy levels). The highest frequency of full check-ins was attributed to pain intensity (183/183, 100.0%), followed by pain interference (175/183, 95.6%), and then mood (152/183, 83.1%). Attrition for check-ins was more common for domains covering physical activity (120/183, 65.6%) and energy (109/183, 59.6%). Note that the sleep domain is only asked once per day regardless of the number of times participants checked in each day (therefore, no denominator provided), which explains the lower number relative to other domains (n=72).

**Table 3:** Individual participant engagement data for the use of the *iCanCope with Pain* app over 7 days.

Participant ID	Full check-ins, n	History views, n	Goals set, n	Library articles accessed, n
1	4	21	2	4
2	8	18	4	8
3	6	3	0	1
4	13	38	11	6
5	7	3	4	2
6	3	4	0	11
7	17	7	2	11
8	7	0	2	7
9	2	0	1	8
10	6	14	2	0
11	7	13	5	1
12	4	0	5	1
13	8	1	1	1
14	5	0	1	0
15	12	36	2	2

A total of 73.3% (11/15) participants completed a total of 158 history views over the week of app use (ie, monitoring their progress over time), with a variable frequency of views for each domain. The highest frequency of domain history views related to pain intensity (51/158, 32.3%), followed by pain interference (24/158, 15.2%), activity (22/158, 13.9%), mood (21/158, 13.3%), sleep (19/158, 12.0%), and energy (18/158, 11.4%).

For the *goals set* feature, 86.7% (13/15) of participants set a total of 42 goals covering 5 areas. The most frequently set goals were for activity (35/42, 83.3%), with fewer for sleep (4/42, 9.5%), energy, mood, and social activity (1/42 for each, 2.4%). Over the week of app use, 63 articles were accessed through the app library by 11 (73.3%) participants. Articles focused on the following content areas (listed most to least frequently accessed; note *n* may be greater than participant numbers, as more than one article could be categorized within 1 content area):

- Making sense of pain (16/63, 25%)
- Sleep hygiene (14/63, 22%)
- Behavioral approaches to pain (coping and mood management; 10/63,16%)
- Activity planning, pacing, and goal setting (8/63, 13%)
- Communicating pain (4/63, 6%)
- Exercising with pain (4/63, 6%)
- Managing fatigue with pain (2/63, 3%)
- Condition-specific care (neuropathic pain; 1/63, 2%)
- Motivation and adherence (1/63, 2%)
- Nutrition (healthy eating habits; 1/63, 2%).

### Phase 3: Qualitative Interview Findings

**Figure 4:**
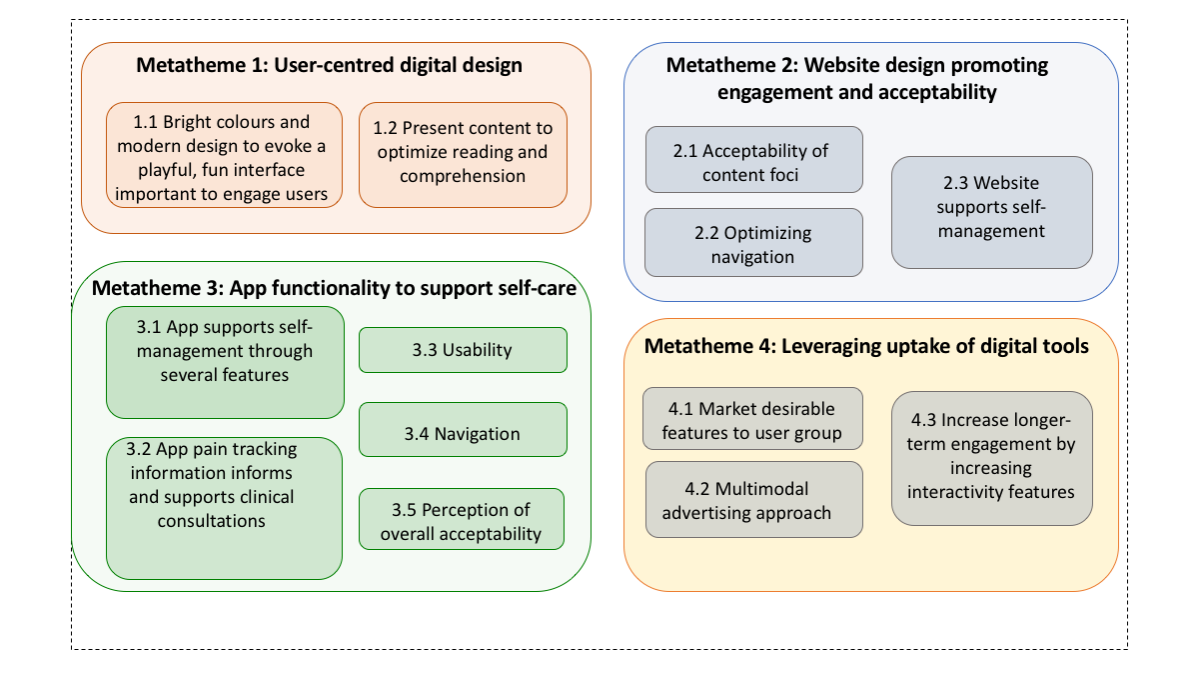
Graphic summary of metathemes and themes derived from qualitative interviews. Metathemes were as follows: user-centered digital design (orange), website design promoting engagement and acceptability (blue), app functionality to support self-care (green), and leveraging uptake of digital tools (yellow).

Findings are presented according to a hierarchical structure aligned to the qualitative data analysis methods, with 4 overarching metathemes identified, supported by themes and subthemes (Figure 4). An overarching summative statement for each metatheme is provided in the text later in the paper. Further details for metathemes, including specific themes and subthemes with supporting quotes, are summarized in Textboxes 4 to 7, respectively. A comprehensive tabulation of all findings is provided in Multimedia Appendix 6.

#### Metatheme 1: User-Centered Design Features Resonating With Young People’s Needs

User-centered design features perceived by participants across both digital platforms as reflecting specific youth-focused digital tools included vibrant color palettes, playful nonlinear shapes, and engaging interfaces that motivated them to interact with the digital tools. Participants described content across both platforms in a manner that was relatable and engaging for young people, with the use of simple language, a variety of formats to deliver information, and chunking of text, rated as preferred features (Textbox 4).

**Metatheme 1: user-centered digital design.**

**Bright colors and modern design that evoke a playful and fun interface are important features of digital tools (website and app) to engage users**

- Vibrant colors associated with being youth-focused

“I felt like the colours were good. I felt like the design was youth-focused, but not necessarily exclusively youth-focused, if you know what I mean? If a 50-something year old were to look at it [the app], they wouldn’t just be like, ’Oh, this is dodgy.’”

Participant number 11; app

- Vibrant colors associated with eliciting positive emotions

“I really enjoyed the bright colour scheme with the blues and the purple and the pinks. I thought it was really calming and really interesting, as well, for a young audience.”

Participant number 9; website prototype 1

- Different shapes, curved edges, lots of images, and illustrations are modern design features that appeal to users

“I really enjoyed how the layout was all the bubbles instead of being just straight text or in boxes, it seemed really interesting and fun.”

Participant number 9; app and website

**Present content to optimize reading and comprehension**

- Use of different formats to deliver information (eg, video, audio, text, illustrations)

“I found it all very easy to read. It was nice big font with a good balance between images to break up the text, as well as videos and stuff like that as well.”

Participant number 3; prototype websites

- Careful attention to font—the size, type, and color all impact readability (particularly if user is feeling unwell/fatigued)

“But the writing itself is a decent size, so for anyone that does have a bit more trouble with that, like if they do have a headache, the fact that it’s a bit bigger is better as well.”

Participant number 1; app and websites

- Using positive, nontechnical language, and less formal wording to enhance engagement

“I guess the thing that struck me the most, I guess, in a good way was how it was sort of almost unprofessional, like it was just asking these questions with almost like childish cartoony images.”

Participant number 3; app

“It’s not ‘for kids,’ there’s no being spoken down to at any point with this site.”

Participant number 10; website

- Short and concise information with links to additional information/external resources

“The shortness of them, the conciseness is pretty helpful as well, so I can just sit down for 5 minutes, read it and then take it on, see if it works and if it doesn’t work, find another one, like, find a method to suit me better.”

Participant number 14; app and prototype website

#### Metatheme 2: Website Design Promoting User Acceptability and Engagement

Participants identified features of the website prototypes that aligned with their preferences for acceptability, ease of navigation, and usability of digital tools. Acceptability here referred mainly to the information being relevant to participants (Textbox 5). Where content was too text-heavy (ie, pain*HEALTH* website), participants suggested modifying density by *chunking information*, using *callouts*, and greater use of supporting images/interactive features (such as video content). Participants liked the use of real-world patient stories on the websites, which resonated with their individual experiences of pain, reduced feelings of isolation, and showed how others had learned to cope with their pain. Navigation features that motivated participants to engage with the website included drop-down menus, clear website layout, simple design, use of a search bar, and minimal clicks to get to relevant content. Participants expressed enthusiasm for a tailored website specific to the needs of young people with pain. Features including meaningful pain management information, practical tips, real-world pain stories, and insights into condition-specific care were positively perceived to support their pain self-management.

**Metatheme 2: website co-design promoting user acceptability and engagement.**

**Acceptability of content foci**

- Information matches user needs

“It’s got plenty of information on different types of pain, things like sleep, which is really helpful as well ‘cos that’s a big part of it, and also information on who you could speak to if you’re having further issues, which is really nice as well because there’s not a lot of that information elsewhere.”

Participant number 11; pain*HEALTH*

“Also, I found that the stories themselves are pretty handy… I think it shows me that I’m not the only one doing it, but they’re also demonstrated methods of coping with it within their everyday life. That was the key bit.”

Participant number 14; pain*HEALTH*

- Information pages too text-heavy and need to be broken up with the use of images, diagrams, and callout text features

“I felt further work could be done to improve things like having a lot more figures and images in the text, so like the articles or explanations, having a lot more diagrams that are explaining some of the science.”

Participant number 22; pain*HEALTH*

- More information wanted on services available and how to access help

“...I think the Help section, where it gave the list of the hospitals and healthcare centres, that needs to be a bit more refined so that it’s easier to see what you were looking for. I’d like to see a website, like a hospital website and then further contact details on that website or instructions on how to get to the details on the service and what they can do.”

Participant number 6; website prototype 1

**Optimizing navigation**

- Drop-down menu feature widely liked and assisted easy navigation

“Everything is easy to find because it’s labelled very nicely at the top there for you and it’s all dropdown, so it’s really easy to go into what you’re looking for.”

Participant number 1; website prototype 1 and 2, version 1

- Website layout design clear and easy to use

“It’s very clearly laid out and really easy to navigate without being, I don’t know, patronising. I’ve been on patronising information sites before.”

Participant number 10; website prototype 1

- Search bar considered an important feature that would assist looking for specific information

“The search bar would make it easier, but it was pretty straightforward and I was able to find what I was looking for.”

Participant number 4; website prototype 1 and 2, version 1

- Preference for minimal clicks to access information

“Probably an update to the home page so when you click on the menu bar you don’t go to another page that’s got all the links there, you actually just go directly to the page that you’re looking for from the menu bar.”

Participant number 14; website prototype 1

**Website supports self-management**

“It’s amazing to think that things like this are coming out because when you first get diagnosed or whatever there really aren’t places like this. So it’s exciting to see that people are starting to think about this. It’s great.”

Participant number 12; website prototype 1

#### Metatheme 3: App Functionality Supporting Pain Self-Management

Participants identified multiple app functionalities that they perceived supported active self-management of their pain. Notably, the daily check-in was seen as encouraging self-reflection and identification of broader factors that may impact their pain, whereas pain tracking information was useful in supporting their pain care through positive behavioral change and guiding clinical pain encounters (Textbox 6).

Usability features that motivated participants to engage with the app included the daily check-in (easy to use), content prompts based on input from check-ins (eg, mood management), and being able to connect with young peers. Participants identified several app functionalities that could enhance daily check-ins, including:

- Ability to set a flexible check-in time that suited their daily routines (versus the standard midday check-in)
- Functionality that allows for mapping output from daily activities and personal diary functionality for reflection/reminders
- Retrospective diary entry capability (in case of missing a day of entry)
- Capacity for entries in different pain areas (ie, pain intensity)
- Alternate mechanisms for ratings at check-in (versus spin a dial)
- Reminder notifications remain active until you have completed a task.

Enhanced functionality to improve self-management included enabling more specific alignment of user goals with measurable outcomes (ie, what goal did you set? did you achieve the goal?) and a metric to map outcome (eg, how much exercise did you achieve?) tailored to the individual. Some participants suggested improved functionality to enable printing and emailing of pain tracking information would be beneficial, as would the ability to personalize an avatar. Participants were largely positive about app navigation functionality, with features indicating strong usability highlighted as intuitive design, ease of use, and accessible content. A majority of participants (10/15, 67%) disliked the app icon *Copey*, indicating that it was more appropriate for younger users. Overall, the app was acceptable to all participants and perceived as a valuable digital tool to promote self-management of pain, with 24-hour access via their smartphones, enabling a flexible fit within their daily routines.

**Metatheme 3: app functionality to support young people’s pain self-management.**

***iCanCope with Pain* app supports self-management through several features**

- Daily check-in encourages self-reflection and identification of broader factors that may impact on pain

“So I think it was good for bringing to light some of those things, because normally you wouldn’t typically finish your day and be like, ‘I was in a lot of pain today. Why was I in a lot of pain?’ and this helps to start those, I suppose.”

Participant number 3

- Pain tracking supports positive pain care behaviors (monitoring, self-reflection, goal setting, coping, and progress over time)

“It was interesting to see how much sleep I got related to my pain and my mood etc... It’s like, maybe I should sleep more.”

Participant number 6

- Encourages setting of goals and monitoring progress

“That was awesome. That part was really good because you can track personally what your goals are, when you’ve completed them, whether they’re short-term goals or long-term goals.”

Participant number 13

**Utilize pain tracking information to inform and support clinical consultations**

“I think it could be used for both, like for just monitoring it yourself. If health professionals need to see how it’s changing, that would be a good tool for them to use.”

Participant number 4

**Usability**

- Daily check-in feature engaging and easy to use

“I just like how easy it is. It doesn’t take very long to check-in and it’s very easy to see how it’s changing and what’s impacting it.”

Participant number 4

- Information interactivity features appealing

“I think after you enter it for three days it starts giving you some recommendations and patterns, which was nice. It gives you feedback and I think it was giving some suggestions of articles, which was good, then you click on them and start reading.”

Participant number 7

- Copey, a divisive character—mixed perceptions regarding the acceptability of the app icon for the target user group

“I quite liked it, but I think it might be a bit too childish for my demographic. The icon is iconic, but I think that’s what is making it childish for the younger group.”

Participant number 6

“I think it is. I think it’s a little childish, but I think that young adults are returning to enjoying more childish things...maybe having the icons be a little bit more adult.”

Participant number 9

- Multifaceted improvements for daily check-in feature

“Yeah, it doesn’t have anything that specifies [what time] I’m checking in and also it seems to let me check in multiple times a day.”

Participant number 10

- Greater functionality (design features) to support end user self-management

“Also with the ‘My Goals’, I think it would be important to link that to your normal check in every day. The information that you provide in your ‘My Goals’ may be relevant to your check in in terms of exercise, sleep, or how you’re feeling today.”

Participant number 7

- Provide different mode settings to enable tailoring the app ‘look and feel’ to each user

“The only thing I would suggest would be a change of background or some personalized settings so people can really make it their own and keep it engaging.”

Participant number 12

- Scales used to rate physical activity need to be revised - not considered intuitive

“Physical activity levels being levelled from ‘great’ to ‘the worst’ doesn’t really make a lot of sense. If it were in terms of minutes, that would, I think, be easier to navigate.”

Participant number 10

**Navigation**

- Easy to use and intuitive

“The navigation around the app was, I felt, really intuitive and really easy.”

Participant number 2

- More guidance on how to utilize functions not related to check-in

“So, I think the main ones obviously that I was using were doing the pain every day, but the other sections I wasn’t too familiar with or understanding well. Yes [more guidance on other sections], because I think the reason I stuck to the first one was because I was instructed how to use it.”

Participant number 15

**Perception of overall acceptability**

- App was considered a valuable resource for monitoring and managing pain

“Yeah, I think the app I would probably use on a daily basis. The app I really, really liked. I don’t know whether I’m still able to use it but yeah, if I’m still able to use it now then I’d love to. I think the app is just amazing.”

Participant number 13

- App readily available using phone

“I thought that was so nice and I wish I had that when I was younger… as I said, it would just be so nice because you always have an information source on your phone and you could just use it whenever you needed it.”

Participant number 9

#### Metatheme 4: Leveraging Uptake of Digital Tools

Participants provided rich insights into how both these digital tools could be leveraged to extend reach into the community and drive uptake by young people with pain. Key themes included marketing the app to potential users, highlighting key functionalities and features that enable tailoring the app to support their self-management (Textbox 7). Testimonials from current users were perceived as marketing opportunities, especially where the testimonial was linked to a social media account identifying a real-world person who had lived experience of pain.

Marketing strategies that included wider promotion through health services or referral by health professionals were perceived by participants as effective ways to increase awareness about these digital tools and their value for self-management of pain. Social media was also advocated as a valuable platform to promote awareness and drive the dissemination of digital tools as well as linking (push) between the app and the (new) website. Some participants suggested promotion via not-for-profit arthritis organizations and educational institutions (schools and universities). To encourage sustained user engagement with digital tools, a few participants recommended a rewards-based design (ie, achieving levels of performance).

**Metatheme 4: leveraging uptake of digital tools.**

**Market desirable features to the user group**

- How the tools can help the user

“I think curiosity drives young people. If you make it available and say that it’ll help you track your pain and so on, I think that might motivate them for people who want to track or monitor what they did.”

Participant number 14; app

- Testimonials from users

“I think people do often read what other people have to say about it, so I think that definitely [testimonials] would make a difference.”

Participant number 12; app and website

**Multimodal advertising approach**

- Referral from a health professional which can be utilized both within and external to the clinical consultation

“…having that a doctors’ surgeries or having doctors or other allied health, like physios or anything like that, refer it.”

Participant number 8; app and website

- Social media advertising

“I think social media could be a big way. Particularly through Facebook or Instagram, having ads that pop up I think could be a good way.”

Participant number 8

- Links through other websites

“But if it popped up on certain websites, like Beyond Blue or something like that as well, because I feel like a lot of them probably go onto those sorts of spaces to look for support... Yeah, because it makes it seem like it is more reliable and a better source of information if it is popping up on something like the government websites.”

Participant number 1

- Promotion through educational institutions

“I think you’ve got to put the information out there and I think one way could be through maybe schools…’If you experience this pain, we’ve got these websites, we’ve got these apps, there are tools available’. I guess the same could go at a university or education system...”

Participant number 8

- Promotion through specific age relevant chronic disease organizations

“In WA, I know there’s a thing called Camp Freedom, which is an arthritis camp with the JIA, but they talk about quite a lot of different apps, they hand out brochures and that kind of thing. So that is a really helpful way to get in as well, because all these kids are dealing with pain.”

Participant number 13

**Increase longer-term engagement with digital tools by increasing interactivity features**

“I think there’s a lot of really valuable information on the website and in the app, but I think it’s that linking to actions and helping to guide people through thought processes, rather than just being information or data tallying. Because it will help engage people for longer with the website and with the services and with the app if they feel like they’re making progress via the engagement with those online and app mediums...”

Participant number 2

## Discussion

### Principal Findings

This study aimed to test the acceptability, usability, and need for adaptation of 2 extant digital technologies to support the needs of young Australians with musculoskeletal pain. Overall, the participants’ perspectives were positive on the acceptability and usability of both the final prototype pain website and the *iCanCope with Pain* app. In using these digital technologies, participants articulated the critical importance of designs being youth-focused, with colorful, fun interfaces that were relatable to promote young people’s engagement in self-management of their musculoskeletal pain. Adaptation of the website prototype was required and informed by rapid cycles of iteration with a focus on improving user engagement. For the app, participant engagement highlighted the value of tailoring capabilities to support individualized pain self-management. Recommendations for app adaptations were modest and primarily related to enhancing functionality (more capacity to personalize the *look and feel*), improved flexibility (check-in time), and capacity to monitor outcomes from goal setting. Participant insights on leveraging these digital technologies to support young people’s self-management emphasized the need for wider promotion, health professional *digital prescriptions*, and strategies to ensure longer-term engagement.

### Strengths and Limitations

Strengths of this 3-phased mixed methods study included the following: (1) adherence to a user-centered eHealth design roadmap [20] reflecting value specifications, co-design principles, iterative cycles of prototype testing, and identification of factors relevant to implementation; (2) evaluation criteria that predict real-world user engagement [20,36,37]; (3) use of an innovative web-based testing platform *fit for purpose*, allowing remote user testing and reach across geographic barriers; (4) rapid cycles of website prototype design, testing, and iteration; (5) use of an analytics platform to evaluate engagement with capture extended over 1 week (user testing often conducted as a short, single session under laboratory conditions); and (6) mixed methods design, using a larger (user testing) sample, thereby providing rich insights on the acceptability and usability of these digital technologies and informing recommendations on the need for adaptation and suggestions to enable implementation.

Limitations included (1) the potential for gender bias given the dominantly female sample; (2) potential participation bias, with recruitment of young people who felt confident in sharing their experiences or who had directly experienced health services/resource gaps; and (3) although our sample is representative of young people from Australia (a developed country with a high-quality health care system), findings are not necessarily transferable to different health care systems, including those of middle or low-income economies.

### Leveraging Digital Technologies to Support Young People’s Self-Management of Musculoskeletal Pain

Leveraging these 2 digital tools (app and web-based) for pain that we have previously co-designed, developed, and evaluated [16,30], enabled us to rapidly create a digital test bed in Australia. Advantages included time and cost efficiencies, maximizing the use of current resources, and avoiding unnecessary duplication [19]. Accelerating this phase of our research program to support young Australian people with musculoskeletal pain [1,2,16] is critical [17,41], given the increasing burden imposed by musculoskeletal pain and the lack of an appropriately skilled health workforce [9,15,17]. Digital technologies such as those we have tested in this study provide an innovative approach to improving timely access to credible and practical self-management for young people with musculoskeletal pain and other chronic noncommunicable conditions [2,42,43], regardless of where they live [1]. This is particularly relevant in the current Australian digital landscape given the implementation of the National Digital Health Strategy [44] and the drive for innovative, sustainable digital technologies to support the implementation of health services and self-care of chronic health conditions [29,44].

### User-Centered Co-Design Critical to Optimizing Acceptability, Usability, and Engagement

Collectively, the findings from our study highlight the importance of user-centered co-design from inception. Our approach is consistent with evidence derived from our recent systematic review of mobile health technologies for chronic noncommunicable disease management in young people, specifically recommendations on effective implementation [2] and the use of an evidence-based roadmap to increase the uptake and impact of eHealth technologies [20]. Our digital tools were perceived as being youth-focused, with a visual design that elicited positive user emotions and promoted active engagement, outcomes aligned with our primary study aims. The positive perceptions of young people on acceptability, usability, and potential engagement are consistent with qualities of product design that predict real-world user engagement with (app and web-based) eHealth interventions [36]. Furthermore, a recent study on design qualities that may predict user adherence to behavioral eHealth interventions in real-world use, found therapeutic persuasiveness (defined as *the incorporation of persuasive design/behavior change principles*) to be the most robust predictor of adherence (ie, duration of use and number of unique sessions), suggesting the importance of persuasive design and behavior change techniques incorporation during the design and evaluation of digital behavioral interventions [45].

Regarding adaptations, the optimization of the app was primarily related to enhancements in functionality (capacity to personalize the *look and feel*, scales for goal setting and monitoring of goals/outcomes, and time flexibility for check-ins). Use of the app over the week varied with individual participant engagement data, highlighting the value of flexible daily check-ins and tailoring capabilities to support individualized self-reflection and monitoring. A similar approach to testing cultural appropriateness, usability, and the need for adaptation of the *iCanCope with Pain* app has recently been undertaken in Norway [32], with preliminary outcomes indicating high levels of acceptability and usability, the only adaptations being the need for optimizing user interaction of the social support feature [32]. The app is currently being evaluated in a randomized controlled trial with a larger sample with chronic pain and juvenile inflammatory arthritis. Other derivatives of the *iCanCope with Pain* app have also been developed for various conditions in children and adolescents, including self-management of postoperative pain [46], sickle cell pain [47], and juvenile idiopathic arthritis [48], with the latter 2 currently undergoing evaluation [46].

Adaptation of the website prototype was guided by rounds of participant feedback, informing rapid cycles of iteration with a focus on improving user engagement. Recommendations for adaptation are mainly related to greater use of chunked information and call outs (key messages in large font that function as calls to action), optimizing navigation (sticky bars, search functions, and drop-down menus), and improving the information on accessing other services and resources to their support care (what services, what they offer, how to access them). These design recommendations were implemented in the final website prototype in preparation for the current (ongoing) phase of finalizing text and audiovisual content in collaboration with young people. This approach to co-design of content will ensure alignment to Australian user needs and preferences [1,14], while also ensuring consistent messaging and self-management domains between the website and the *iCanCope with Pain* app.

Operationally, the intention for future use of these 2 digital platforms in Australia is to offer complementary digital tools with different functions that can interact bidirectionally (eg, by use of push notifications). Such an approach offers flexibility in supporting both individualized tailored self-management (app), while concurrently providing the capacity for richer audiovisual content and resources specifically codeveloped with and for young Australians (website) and critically, explicitly linked to Australian health services and systems [2]. On the basis of our impact evaluation of the adult pain*HEALTH* website [30], digital platforms can operate as an important health strengthening tool [49], linking consumers/caregivers with their clinicians, services, and systems. We have also demonstrated that this approach promotes more holistic integrated pain care models [17,30] and enables consistency of messaging between consumers and their clinicians (eg, sharing of short targeted audiovisual content during clinical consultations about other people’s pain experiences, and how they have implemented positive evidence-based behaviors to improve their pain care). The web-based platform will also include contemporary, evidence-based condition-specific musculoskeletal knowledge (eg, about low back pain and juvenile inflammatory arthritis) with links to best practice pain self-management (eg, making sense of pain, pain education, coping skills and behavioral approaches to pain, encouraging movement, activity and exercise with pain (pacing), appropriate use of medicines) [16,50]. Additional advantages of a web-based platform include the capacity for real-time updating at lower resourcing and cost than app-based technologies, multiple-platform compliance (ie, capacity for use on various devices), easy access, and linking to other entities (eg, consumer, tertiary educational, and health professional bodies). Advantages of the app included empowering young people to take *their health in their hands*, supported by individual tailoring abilities of the app, informed by check-ins, with self-monitoring and self-reflection supporting helpful habits (behavior change) and complementing clinical care.

### Conclusions: Insights and the Next Steps

Outcomes from current, ongoing trials of the effectiveness of the *iCanCope with Pain* app will help to inform our decision on the most appropriate approach to evaluate the implementation of these digital technologies in Australian pediatric pain care settings. We envisage the use of a contemporary and flexible evaluation approach that moves beyond traditional effectiveness designs to consider alterative hybrid trial designs [51] or multidimensional and whole-of-system evaluation approaches, such as a *benefits evaluation* [52] that is best aligned to the Australian digital ecosystem and positions these digital tools for real-world implementation [51]. The adaptation of user-centered design and implementation science methods from inception (such as done here) can mitigate the risk of low rates of implementation and the associated research waste [19]. In this context, we gained valuable insights from young people to support and enable implementation, including taking a *whole-of-health* (systems, services, and clinical-level) approach to facilitate real-world dissemination and embedding. These findings are in accordance with recommendations from our recent systematic review of mHealth technologies for noncommunicable chronic disease management in young adults [2].

Digital technologies are positioned to enable the rapid transformation of health care, with their critical role highlighted by the release of the first World Health Organization guideline establishing recommendations on digital interventions for health system strengthening [53], and more recently, local recommendations for transforming health in Australia [54]. The outcomes from this study are, therefore, timely considering the reorientation of health services and system reform toward better integrated management of chronic health conditions [55], healthy aging across the life course [56], and the expectations of young people with chronic musculoskeletal pain for access to evidence-based digital tools to support their self-management [1]. Although we are cautious and thoughtful about the many broader research questions that remain on the evidence for the use of digital technologies in health care innovation (consumer needs and preferences; synergy with current services and systems; capacity to interface with current health systems, services, and workflows; achievement of broader system goals; and stability and sustainability), we suggest that this should not be a reason to accept the current default position in Australia, where many young people with musculoskeletal pain do not have timely access to reliable evidence-based services and resources that promote and support helpful behaviors and improved well-being.

## Appendix

Multimedia Appendix 1 Consolidated criteria for reporting qualitative studies: 32-item checklist.

Multimedia Appendix 2 Phase 1: website user testing guide.

Multimedia Appendix 3 Phase 2: iCanCope with Pain app user testing guide.

Multimedia Appendix 4 Phase 3: interview schedule.

Multimedia Appendix 5 Phase 1: website user testing criteria-based group-aggregated outcomes.

Multimedia Appendix 6 Phase 3: comprehensive summary of metathemes, themes, and subthemes with supporting quotes derived from qualitative interviews.

## Abbreviations

APEEE: analytics platform to evaluate effective engagement
eHealth: electronic health
ÖMPSQ-SF: Örebro Musculoskeletal Pain Screening Questionnaire—Short Form
