# Supplementary material for: Evaluation of Digital Technologies Tailored to Support Young People’s Self-Management of Musculoskeletal Pain: Mixed Methods Study
Source: J Med Internet Res. 2020 Jun 5;22(6):e18315. doi: 10.2196/18315 (PMC7305555; doi:10.2196/18315)
Supplement: Multimedia Appendix 1 [file jmir_v22i6e18315_app1.pdf]

Multimedia File 1

Consolidated criteria for reporting qualitative studies (COREQ): 32-item checklist [1].

**No. Item**	**Guide questions/description**	**Addressed in manuscript / response**
**Domain 1: Research team and reflexivity**		
Personal Characteristics		
1. Interviewer/facilitator	Which author/s conducted the interview or focus group?	Reported in Reported in “Methods” p14
2. Credentials	What were the researcher’s credentials? E.g. PhD, MD	Noted on title page
3. Occupation	What was their occupation at the time of the study?	Reported in “Data collection” p14
4. Gender	Was the researcher male or female?	Female
5. Experience and training	What experience or training did the researcher have?	Reported in “Methods” p14
Relationship with participants		
6. Relationship established	Was a relationship established prior to study commencement?	Reported in Reported in “Methods” p8
7. Participant knowledge of the interviewer	What did the participants know about the researcher? e.g. personal goals, reasons for doing the research	All participants were provided with a detailed Participant Information Statement that outlined the study team, the background to the study, aims, and requirements for participation. Participants were also briefed about the interviewers’ background in phone contact prior to the scheduled interview. Reported in Reported in “methods”
8. Interviewer characteristics	What characteristics were reported about the inter viewer/facilitator? e.g. Bias, assumptions, reasons and interests in the research topic	Reported in Reported in “methods” p14
**Domain 2: study design**		
Theoretical framework		
9. Methodological orientation and Theory	What methodological orientation was stated to underpin the study? e.g. grounded theory, discourse analysis, ethnography, phenomenology, content analysis	Reported in “Data analysis” p15-16

Participant selection		
10. Sampling	How were participants selected? e.g. purposive, convenience, consecutive, snowball	Reported in methods: "Recruitment and sampling" p8
11. Method of approach	How were participants approached? e.g. face-to-face, telephone, mail, email	Reported in "Recruitment and sampling" p8
12. Sample size	How many participants were in the study?	N=15 participated
13. Non-participation	How many people refused to participate or dropped out? Reasons?	Reported in "Results". Five people who were invited to take part declined to do so. Reasons were provided in results as to why they did not participate p 17
Setting		
14. Setting of data collection	Where was the data collected? e.g. home, clinic, workplace	Reported in "Methods" p14 (remotely)
15. Presence of non-participants	Was anyone else present besides the participants and researchers?	No. Reported in "Methods" p8, 15
16. Description of sample	What are the important characteristics of the sample? e.g. demographic data, date	Reported in "Results" p17-18
Data collection		
17. Interview guide	Were questions, prompts, guides provided by the authors? Was it pilot tested?	Reported in "Methods" p14. The interview schedule is provided as a Multimedia File 4
18. Repeat interviews	Were repeat interviews carried out? If yes, how many?	No
19. Audio/visual recording	Did the research use audio or visual recording to collect the data?	Reported in "Methods" P14
20. Field notes	Were field notes made during and/or after the interview or focus group?	No
21. Duration	What was the duration of the inter views or focus group?	Reported in "Methods" P14
22. Data saturation	Was data saturation discussed?	Yes. Reported in "Data Analysis" P15-16
23. Transcripts returned	Were transcripts returned to participants for comment and/or correction?	Yes. Reported in "Methods" p14

Domain 3: analysis and findings		
Data analysis		
24. Number of data coders	How many data coders coded the data?	Reported in "Data analysis" p15 (N=1 primary coder; n=2 addition reviewers of coding; and 5 transcripts externally reviewed and coding validated with n=2)
25. Description of the coding tree	Did authors provide a description of the coding tree?	No. See Multimedia File 6 for complete coding framework, with metathemes, themes and subthemes
26. Derivation of themes	Were themes identified in advance or derived from the data?	Themes were derived inductively and then deductively followed by an iterative process of grouping codes into concepts, reviewing transcripts and further refining themes. Reported in "Data analysis" P15-16
27. Software	What software, if applicable, was used to manage the data?	No
28. Participant checking	Did participants provide feedback on the findings?	Yes. p14
Reporting		
29. Quotations presented	Were participant quotations presented to illustrate the themes/findings? Was each quotation identified? e.g. participant number	Yes. Reported in "Results" And Supplemental File 6
30. Data and findings consistent	Was there consistency between the data presented and the findings?	Yes
31. Clarity of major themes	Were major themes clearly presented in the findings?	Yes
32. Clarity of minor themes	Is there a description of diverse cases or discussion of minor themes?	Yes

1. Tong A, Sainsbury P, Craig J. Consolidated criteria for reporting qualitative research (COREQ): a 32-item checklist for interviews and focus groups. Int J Qual Health Care. 2007;19(6):349-57.
