# Supplementary material for: Evaluation of Digital Technologies Tailored to Support Young People’s Self-Management of Musculoskeletal Pain: Mixed Methods Study
Source: J Med Internet Res. 2020 Jun 5;22(6):e18315. doi: 10.2196/18315 (PMC7305555; doi:10.2196/18315)
Supplement: Multimedia Appendix 2 [file jmir_v22i6e18315_app2.pdf]

pain*HEALTH* user testing

Hi ______________________________,

Thank you again for taking the time to participate in this study. This should only take up to 10 minutes of your time. Before we begin, I'm going to give you a brief overview of the test and how it will work.

This session is pretty straightforward — I'll be giving you a broad task to complete and then asking questions as we go along. Before I tell you the task, I'll be giving you a little bit of context behind it.

It's really important to know that we are only testing the site, not you. You can't do or say anything wrong here. Please feel free to let me know at any time if there's something you like, dislike, if you're confused, etc...

Also, I'd like you to "think aloud" as much as possible. By that, I mean that I'd like you to speak your thoughts as often as you can. For example, you may be looking at a page, suddenly see something you didn't see before and want to click on it. In that case, saying something like "this caught my eye so I'm going to see what it is" would be very useful.

If at any point you have questions, please don't hesitate to ask. Do you have any questions so far?

Ok, let's get started.

Scenario 1 – Find and watch Daniel's pain story

Background

You have just arrived onto the painHEALTH website as you have been told about it as a helpful resource to assist you in managing your musculoskeletal (muscles, joints and bones) pain care.

You wish to watch and read about other people's experiences with their respective MSK pain condition.

Task

Find 'Daniel's pain story' and hear about his story

Questions (Gauge feedback) [navigation; content; user engagement; therapeutic persuasion/alliance; expectations]

- Did you have any trouble finding this page?
- What would assist you to finding this page? [skip if above answer is no]
- Did you find the type of content we used to tell Daniel's story to be engaging? If yes, what was engaging? If no, what would make it more engaging?
- Are there any other formats / types of content that could enhance telling Daniel's pain story?
- Any other general feedback that would improve the website for you?

Feedback / Notes

Scenario 2 – Find and complete the Orebro pain self-check questionnaire [navigation, usability, user engagement, improvements/enhancement]

Background

Since learning about Daniel's pain story, you wish to complete a pain self-check questionnaire to understand more about your pain.

Task

Find, start and complete the 'Orebro Musculoskeletal Pain Screening' self-check. This self-check is used to help identify who might be at risk of pain persisting and to guide advice and care.

Questions (Gauge feedback)

- Did you have any trouble finding this page?
- What would assist you to find this page? [If no to above, skip this]
- Did you find the pain self-check a useful tool?
- Could anything improve the self-checks?
- Are there any other formats / types of content that could enhance telling Daniel's pain story?
- Any other general feedback?

Feedback / Notes

Scenario 3 – Find and read the 'Making Sense of Pain' management module [navigation, user engagement, therapeutic persuasion/alliance, content]

Background

After completing a pain self-check, you now wish to learn more about your pain and how to make sense of pain.

Task

Find and read the 'Making Sense of Pain' management module

Questions (Gauge feedback and

- Did you have any trouble finding this page?
- What would assist you to finding this page? [If no to above, skip this]
- Did you find the type of content to be engaging? If yes…what was engaging. If no, what would make it more engaging?
- Are there any other formats / types of content that could enhance this pain module?
- Any other general feedback/ideation?

Feedback / Notes

Scenario 4 – Further assistance [navigation, usability, expectation)

Background

You wish to reach out or speak to someone about how you find further assistance for your pain (where you can get more help, who you can see, where services are provided)

Task

Find the 'Further Assistance' page as if you wish to contact 'Arthritis and Osteoporosis WA'

Questions (Gauge feedback and

- Did you have any trouble finding this page?
- What would assist you to finding this page? [If no to above, skip this]
- Did you find the type of content to be useful?
- What type of content / information would you expect to see on a page like this? Was anything missing
- Any other general feedback/ideation?

Feedback / Notes

Final feedback

That completes our user testing. You feedback is invaluable and will be used to help us determine how we build our newest iteration of this website in the future.

Based on what you've seen today, do you have any overall feedback about the website. For example:

- would you see yourself accessing/utilising this website if you needed assistance?
- did you have any likes you liked/disliked about the site?
- what additional features/functions would you like to see in the future that could further assist you in managing musculoskeletal pain?

Thank you and follow up

Thanks for your time.

Are you happy to be contacted for any future user testing we wish to perform once we are closer to a final version of the website?

Would you be interested in testing a pain 'app' for a week?
