# Supplementary material for: Evaluation of Digital Technologies Tailored to Support Young People’s Self-Management of Musculoskeletal Pain: Mixed Methods Study
Source: J Med Internet Res. 2020 Jun 5;22(6):e18315. doi: 10.2196/18315 (PMC7305555; doi:10.2196/18315)
Supplement: Multimedia Appendix 3 [file jmir_v22i6e18315_app3.pdf]

Multimedia File 3. Digital Technologies for Young People with Musculoskeletal Pain: iCanCope with Pain user-testing

About the iCanCope with Pain App
The iCan Cope with Pain program is an integrated website and app that was co-developed with young people, clinicians and researchers at SickKids Hospital and the University of Toronto, Canada.

The aim of using iCanCope is to help you to recognize, understand and better manage your pain and functioning.

In collaboration with colleagues in Canada, we are testing the program with young people in other parts of the world, including Ireland and Australia.

There are two main features, which are designed to work together:

a. iCanCope with Pain app (for iPhone and Android)
b. iCanCope with Pain website

The app has tools to help you track your symptoms, set goals and connect with others. The website has in-depth information, videos, animations and audio clips.

The **iCanCope with Pain app** is a resource that you can always have in your pocket to support you in your daily life. Use it to:

- keep track of your pain and function (for example, tracking your sleep, mood, physical activity and energy levels)
- set and track realistic goals about things that matter to you and will help improve your functioning
- access tools to cope with pain in your daily life (exercising with pain, managing mood, communicating about pain)
- connect with other young people who have chronic pain.

This **iCanCope with Pain** website has 10 sessions and provides more in-depth information for when you have questions about chronic pain. Check out the sessions that are relevant to you so you can find the right approaches to manage your pain.

Use it to:

- Learn more about your chronic pain and how you can manage it
- Understand the importance of combining physical, psychological and pharmacological (medications) strategies to manage your chronic pain
- Access videos and animations to better understand key points and hear from other people living with chronic pain
- Access audio clips to guide you through meditation and relaxation exercises.

We will collect some information about your use of the program to see if it is working for you. All your information is kept private and confidential and is de-indentified.

On the next 2 pages, we will guide you through getting up and running with the app.

Multimedia File 3. Digital Technologies for Young People with Musculoskeletal Pain: iCanCope with Pain user-testing

What do I need to do to set up the app?

Steps to set up user account:

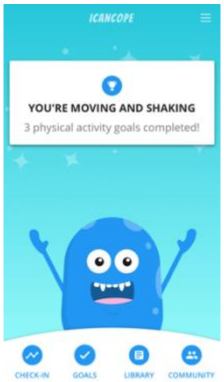

1. Go to the iTunes App store
2. Select the **iCanCope with Pain app** – please make sure you choose the right app, as there are different versions of the iCanCope app for other conditions!
3. Download the app onto your personal device
4. Using your allocated user code 'austest1**' create an account by selecting the menu top right of the screen. This user code is a unique number allocated to you only. Then enter the password 'testuser1' you have been allocated.**
5. You can explore the app function and use the app as needed over the 1-week study period

Here are 4 quick tasks for you to get up and running:

Task 1. About me

Here you are setting up the app for your personal use. All data is anonymous and is de-identified. Set up your user profile:

> 'About me' – you can add details such as name (whatever alias you want), age, gender, school or work
> 'Pain areas' – you can select which areas you have pain in touching the body chart avatar. Select as many areas as you need to – you can add these separately or as a whole if the areas are all linked. You can flip the body chart too so that you have front/back of the body.
> Hit 'continue' and select as many pain descriptors as you like.
> Hit continue and select the 'triggers' for your pain

You will see on this same page 'History'. Touching on any one of these will pull up your tracking data. Once you have set goals and done your daily check ins, you can track your daily data on the calendar for each of the following:

- Pain Level
- Pain Interference
- Mood
- Sleep
- Physical Activity
- Energy

Multimedia File 3. Digital Technologies for Young People with Musculoskeletal Pain: iCanCope with Pain user-testing

Task 2 Setting Goals to help you improve pain and do the things you want to do.

Setting structured goals is one of the key skills that help you better manage pain. You can set goals in 5 main areas:

1. Physical activity
2. Sleep
3. Social
4. Mood
5. Energy

Let's use 'Physical activity' as an example:

> At the bottom of the screen, select the 'Physical Activity' goal icon.
> Next, touch the goals 'icon'
> You will be taken to a screen where you can set your own goals
> Rotate the 'goal wheel' to the goal area you want set
> Hit the 'continue' button
> A screen will pop up asking you to create a goal using the '+' symbol
> Type the goal into the text field eg 'walk 2 kms' and hit 'Continue'
> You can use the alarm symbol you can set a date and reminder for achieving the goal eg for your run 2 km run, the target date may be in 2 days' time
> When goals are completed they are listed under the 'completed' section in the app.

Task 3. Please complete a daily check-in a day for 7 consecutive days.
See how you're progressing by self-monitoring your daily pain levels, pain interference, sleep, mood and physical activity.

The app should push a daily reminder notification to you about these items:

- Rate your pain from 1-10
- Rate how pain limited your activity levels ('way less' through to 'way more')
- Rate your mood ('great' to the 'worst')
- Rate your physical activity level ('great' to the 'worst')
- Rate your energy level ('great' to the 'worst')

Task 4. The library and the community

The library is where you can find resources that are designed to support you better manage pain.

These short articles cover just about everything from nutrition to mood, sleep, work and study, social activities, self-worth, coping with pain, dealing with setbacks, talking to friends, employers and teachers about pain and lots more.

You can check out the 'Community' section, but **please do not post any comments** as the app is currently under trial conditions in Canada.
