# Supplementary material for: Evaluation of Digital Technologies Tailored to Support Young People’s Self-Management of Musculoskeletal Pain: Mixed Methods Study
Source: J Med Internet Res. 2020 Jun 5;22(6):e18315. doi: 10.2196/18315 (PMC7305555; doi:10.2196/18315)
Supplement: Multimedia Appendix 4 [file jmir_v22i6e18315_app4.pdf]

Evaluation and monitoring of consumer oriented resources for the self-management of musculoskeletal pain

Consumer interview schedule

<u>Introduction</u>

Thank you for agreeing to participate in this study. The purpose of the study is to discuss your experience of using the pain*HEALTH* and iCanCope with Pain – [choose appropriate one, or both as relevant] digital resources resources.

We are really interested to understand how you used the [pain*HEALTH* website and the iCanCope with Pain app], what you have found most useful and what you think can be improved. We are also interested in knowing how you use these resources to support your management of pain and your general wellbeing.

Before we start, I just want to check that you are comfortable with me recording our discussion? I use the recording to obtain a transcript of the discussion to help me with writing up the findings of this study.

I am the only one who handles the transcript and I will send you a copy of the transcript to make sure you are happy with the information and for you to see that everything is anonymous and that you won't be identified in anyway.

Just to reassure you that all the information discussed today is strictly confidential. I'm really interested in hearing your experiences and thoughts about these digital tools, and no detail is too small and there are no right or wrong answers.

Also, if you don't feel comfortable talking at any point, please let me know and we will stop. Please also let me know if you need to stop to move around or change position if required. You are also free to pull out of the study at any time.

[start audio recording]

Background/context

1. Have you used digital tools before for your pain management?
    a. [prompt] what tools? What apps?
2. Where do you get most of your information/practical guidance about your pain management?
    a. [Prompt] Health professionals/organisations/internet/friends and family?
    b. [Prompt] Which do you consider the most trustworthy sources to help you manage your pain? And why? (i.e. can they identify what is reliable?)
    c. [Prompt] If internet – how often seek health information or support about pain
    d. What type of information, advice or guidance do you look for (i.e. not just info but practical skills or things they can do)?
    e. Has that information/practical skills been helpful overall
    f. [Prompt] If internet - what devices do you use (computer/tablet/phone)?

painHEALTH website

3. How was your experience using the painHEALTH website?
    a. What worked well?
    b. Is it suitable for young people?
    c. If not, what would you change?

***iCanCope* App**

4. How was your experience using the 'app'?
    a. What worked well?
    b. Is it suitable for young people?
    c. If not, what would you change?

Digital tools

5. Have you come across or used any other digital tools to help manage your pain?
    a. [if yes] Can you describe these?
    b. [if yes] Do you still use these other tools/resources?

6. Can you discuss your overall impression of these digital tools (are they helpful? Age-appropriate? Engaging? Meaningful to you, acceptable, makes sense, useful, usable, easy to navigate, good content, etc)?

7. In your opinion, how easy is it to find the information/resources you want/need within each digital tool?

8. Thinking about these digital tools, what things do you find (or have you found) most useful for helping you manage your pain?
    a. [Prompt] This can relate to a specific piece of information or feature on the website (pain stories, self-checks, fact sheets, video clips, mp3 files etc; goal settings, library)
    b. Why/how have these been useful?

9. When you have visited these digital tools, which aspects do you most use? (condition, pain stories, management, self-checks, further info; goal settings, library, checkin etc)
   [For each section]
    a. What is most helpful about this section **for you**?
    b. Do you use this information/resources as part of managing your pain? If so, how?
    c. Does any of the content help you to manage your general health more broadly?
    d. Is there anything you feel that is missing that could better assist you in your pain management, or be presented differently to make it easier for you?

10. Self-checks can assist users to identify any potential serious pain conditions that may require medical attention. Have you used any of the self-checks in either tool?
    a. [if yes] Which one(s) and did you find the self-check useful?
        i. [if yes] In what way was it useful for you?
        ii. [if no] Can you tell me why you didn't find it useful?

11. Do you think we need to re-design either tools and what changes would you make?

12. Is there anything else that you would like to tell us about these digital tools?

13. How would see this being implemented to ensure uptake and engagement?
