# Supplementary material for: Evaluation of Digital Technologies Tailored to Support Young People’s Self-Management of Musculoskeletal Pain: Mixed Methods Study
Source: J Med Internet Res. 2020 Jun 5;22(6):e18315. doi: 10.2196/18315 (PMC7305555; doi:10.2196/18315)
Supplement: Multimedia Appendix 5 [file jmir_v22i6e18315_app5.pdf]

Multimedia File 5.

**User group**	**User design criteria**	**Insights and supporting quotes**
Group 1		
	Navigation	Enhancing navigation: compared to the control pain*HEALTH* website, the use of menu 'dropdowns' to navigate the desktop prototypes enhanced the users' experiences and improved the ease of completing tasks:<br>*"I like the use of the dropdowns. Made it much easier to find the pages."*
	Usability	Enhancing functionality: Users agreed that the self-checks were useful. In both prototypes, the use of having radio buttons 'grouped' made the user input easier to scan and interact with (tap/click).<br>*"I don't know why, but the other prototypes made it seem easier to click on and scan when wanting to complete the quiz."*
	User engagement	Content presentation and relatability: 'Further Assistance' [baseline website] had a clearer meaning than the use of the wording 'Further Support' used in both prototypes. When asked further if this resonated with the type of content users saw on our baseline website, users indicated that this terminology did not align with their expectations. Suggestions from users of terms that would better reflect their expectations of what they would interact with included: 'further contacts' and 'contacts' or 'help'.<br>*"I get confused with Support and think it's more aligned to tech support." "When I hear the term Support, I think of live chat and other tech things."*
	Content	Level of literacy: Overall pain modules and the content was very well received. Some users reported that they would prefer some of the more complex words or concepts to be explained more easily with the use of a glossary page and illustrations:<br>*"I'm more of a visual learner. Being able to see what a pain jigsaw is in an illustration or having a tooltip or glossary of what certain terms mean like 'nociceptor'".*

Multimedia File 5.

	Acceptability	Expectation and Motivation: Users identified that they wanted to know how the self-check results could help them, what they could do with these results, what health professional or specialist should they contact and was there other supportive content available on the website?<br><br>*“Yeah it’s useful, but what do I do next? How do I use these results?”*<br><br>Likability: Overall, users leaned more strongly towards preferring the ‘round and bright’ prototype variation, with only 2 users having a preference to the square/geometric variation.<br><br>*“I like the roundy one. It makes it feel more friendly and approachable rather than all square and sharp.”*<br><br>When asked why they preferred the ‘round and bright’ version, they explained this related to the look and feel of the website, with use of big yellow/pink colours and shapes (from the geometric prototype) making the page a bit “noisy”:<br><br>*“Thinking if I was 15. I wasn’t sure if this website was aimed at me or rather 10 – 12 year old’s.”*
Group 2		
	Navigation	Compared to the baseline website, and prototype iterations implemented to enhance a user’s navigation to Daniel’s story, were well received with the additional dropdowns making for easier navigation:<br><br>*“I like this short summary - dropdown. These links look more inviting [i.e. than baseline website]. I like this - 3 minute read time. More engaging and easier to find. Really liked the little summary to help me choose a pain story”.*<br><br>*“No issues - found this very easily. Ah, it's pretty cool. I think the quote engage the reader. It's like their a real person”.*<br><br>*“You can really see the main points of the story. Liked the link back to management modules for more relevant information”.*

Multimedia File 5.

	Usability	Functionality: Overall, while functionality for the prototype was more positive for the prototype compared to the baseline website, users perceived that the results page still needed more specific content to empowering users to make sense of the results and direct them to the right channels to better access the right help.<br><br>*"I'd like to understand what my score means, what can I do with it and what can I learn about my condition/score now?"*<br><br>*"Maybe include some questions you could discuss with a health professional about your results."*<br><br>*"Putting in your total score can be quite funny… You could be either worse or better than you thought."*<br><br>The use of a progress bar rather than a step-wise navigation was preferred by users when completing the pain self-checks:<br><br>*"I think the progress bar is good. It's telling me how much I've completed."*<br><br>*"It makes it feel more like a quiz than steps in a process."*<br><br>To further enhance the experience progress bar and percentages were recommended.<br><br>The use of email functionality to on-forward the self-check results to users was perceived positively:<br><br>*"I clicked on email results immediately. It means I can send them to myself and have them on hand whenever I need them."*<br><br>Informing a user what the self-check is and how it can help them, was positively perceived, setting their expectations for the length of time taken to complete the self-check and use the results:<br><br>*"I liked the starting screen…It told me how long it's going to take which was great."*

Multimedia File 5.

<table>
<tr><td></td><td></td><td>“It’s good it told me what the self-check does and how it could help me.”</td></tr>
<tr><td></td><td>User engagement</td><td>When users read and interacted with the content in both ‘Daniel’s pain story’ and in the ‘Making Sense of Pain’ management module, they reported the mix of content types and delivery modes (text, video clips, audio) was highly engaging, relatable and relevant for young people:

“This is a very affirming four paragraphs of text”

“That’s really what you need to hear and want to know”

“I really liked the videos. When my pain is really bad, it is quite hard to read. However, it’s easier to watch then.”

Users discussed how the hyperlinks within the websites are useful, however navigation could be improved.

“It’d be good to be able to show when you’re linking off to an external website.”

“By clicking on these links, it takes me back to another page. It’d be good for these to open in another tab so I don’t lose where I was.”

One user touched on how the use of social media (Instagram) or a forum could be helpful to assist a user to communicate with pain story individuals and to seek advice, see how they’re living with pain:

“Social media or a forum where people can have conversations about what worked for them, help other people get in contact with one another.”

“If you could get health professionals on the forum too that would be great.”</td></tr>
</table>

Multimedia File 5.

	Content	Users articulated that breaking up management modules into 'chapters' would help users to read the content in more digestible 'bite-size' chunks supported by the use of visuals/video chunks.<br><br>*"Break down the content into 'chapters' / snippets if you'd like to help someone younger digest the info. Kind of like a bit-size snippet, like Instagram stories."*<br><br>*"Suggested maybe shortening length of content… Depending on my mood, the length might make me leave. If I'm procrastinating I'd leave. However, if I genuinely looking for help, I'd prefer one page."*<br><br>Compared with the baseline website, for the prototype presenting content using a block quote from Daniel's pain story with a link to the management module was well received, making the content easier to read and relate to:<br><br>*"I like these quotes."*<br>*"It makes it feel more personable."*<br>*"It makes the stories sound like they're from real people."*<br><br>The 'Further Assistance' page made sense to most users. However, when asked to explain their understanding of this term further, users described their expectations for specific assistance to address their individual musculoskeletal pain queries, rather than contact phone numbers for services.<br><br>*"Further assistance makes me think of a tangible assistance rather than contact number."*<br><br>Including the categories of the types of support services available and listing these in alphabetical order aligned much closer to user expectations and preferences.

Multimedia File 5.

	Acceptability	Enhancements made to the dropdowns for pain stories and management modules with the inclusion of 'category' chapters (indicating nature of content) were well received amongst all users.<br><br>*"I like the dropdowns."*<br><br>*"Ah wow – cool."*<br><br>For the prototype website, use of drop down menus with icons to identify specific pain management content/practical tips and skills were well received. This included content such as: learning about pain; neuroplasticity; pain types; making sense of pain; approaching pain; pacing and goal setting; movement with pain; mindfulness and pain; yoga and pain; sleep and pain).<br><br>*"That's heaps easier. I like the sections. Helps break up the content easily."*<br><br>One user had a strong negative response to the 'mindfulness' category:<br><br>*"Mindfulness makes me feel less likely to click on the management module. Strategies could be more appropriate – It's not meditative or spiritual things. It's more concrete things and how you can put things together in your head about it (pain)."*<br><br>The size of the images on the baseline website caused one user anxiety and confused them about the type of content:<br>*"I had some trouble finding the making sense of pain management module. I think it's because of the pictures. The pictures just make me think that these are stories of different people."*<br><br>For the prototype website, using images that reflected what the management modules were about rather the photos of people better explained and showed users what to expect:<br>*"Ah, much better. I feel calmer on this site. Some of the expressions of the other photos were giving me anxiety."*

Multimedia File 5.

		"I really like this version, it's brighter, the text is bigger so easier to read"<br><br>*"I like the curvy font and rounder buttons. The colours and palette are really engaging."*<br><br>*"I like this site a lot compared to the other one. It makes me calmer."*
Group 3		
	Navigation	Users indicated strong support for the prototype website navigation enhancements with no issues reported.<br><br>*"I like the dropdowns - especially the stories. It gives you a background about the story and the name is easy to find before you click."*
	Usability	Self-check functionality enhancements were well received by users:<br><br>*"Had a little smile once found results. 'Yeah this is better - It's more what I was expecting than the other one [baseline website]."*<br><br>*"Cool splash screen for the quiz…liked the graphic design of the scores…it's probably the most optimistic way of showing the results."*<br><br>*"It's great you can email your own results or get further contacts immediately. It's good it explains what the results mean, even if it's good or bad"*

Multimedia File 5.

		One user perceived that a prototype enhancement would be the ability to 'read the pain story out aloud' in case a user is in pain or they wish to save their laptop battery.
	User engagement	Users were very positive about the prototype website and suggested there should be more websites that are an 'aggregate' of pain management (i.e.; a holistic, integrated approach) to make it easier for a user to find, research and understand their individual conditions of pain from the one website.<br><br>Users suggested that the use of more illustrations within the management modules, could help to support and explain content. While videos were perceived as supporting users, having illustrations could also help break up the text sections to improve readability and user engagement.<br><br>*"Really engaging content. Liked the background of the story for Daniel's case. Liked the pull quotes".*
	Content	Overall content was very well received by users, with the use of "pull" quotes and suggested read times, specific features appealing to users:<br><br>*"Format and content was really good."*<br><br>*"Liked the '3 minute read' meta info".*<br><br>*"There's a lot of terms in the content, however they're explained simplify and clearly. Liked the pull quotes."*<br><br>For the 'Further Contact' page, the introduction of 3 navigation tabs resulted in some users initially getting a bit lost on the page (~2 - 3 seconds) until users cognitively processed the functionality of the tabs. Users had no issues with finding data as it was presented within the page and is was reported as

		logically ordered (alphabetically) and easy to scan. One user suggested the use of a side bar section that can act as an 'anchor link' that is sticky and follows the user up/down the screen and scrolls their viewpoint to the relevant contact area could further assist finding relevant contacts.
	Acceptability	Optimisation of the prototype palette was well received, with users reporting the website look and feel as engaging, fun and appropriate to their demographic. The '3 minute' reading time to set expectations and the further reading articles was highlighted by users as supporting their needs.<br><br>The increased size of font in this prototype was highlighted by users as easier to read, rounder and "friendlier" than the baseline website.<br><br>*"I definitely like the prototype a lot better [than the baseline website] - as a teenager itself. The pictures on the site make it feel teenage friendly"*<br><br>*"Even having the images on the sides makes it more teenage focused"*<br><br>*"Really like the graphic design and the font"*<br><br>*"Really liked the real stories, helps you see how real people have managed their pain"*<br><br>*"Yeah, that's amazing. I really like the colour palette and how it suggests your reading time."*<br><br>*"Like the bolding and use of icons for the helpful insights takeaway".*
