# Supplementary material for: Evaluation of Digital Technologies Tailored to Support Young People’s Self-Management of Musculoskeletal Pain: Mixed Methods Study
Source: J Med Internet Res. 2020 Jun 5;22(6):e18315. doi: 10.2196/18315 (PMC7305555; doi:10.2196/18315)
Supplement: Multimedia Appendix 6 [file jmir_v22i6e18315_app6.pdf]

META theme 1: USER-CENTRED DIGITAL DESIGN	
**Key theme 1.1: Bright colours and modern design that evoke a playful and fun interface are important features of digital tools to engage users**<br>Across both digital tools, participants identified key design features that were considered important to appeal to younger users, namely bright and vibrant colours, and playful interface using non-linear shapes and images. These features are discussed in further detail below.	
Subtheme 1.1.1: Vibrant colours associated with being youth focused<br>Participants perceived that bright colours were associated with being more 'youth focused' because they were fun, attention grabbing and more engaging. In particular, users contrasted the bright prototype *painHEALTH websites* which was considered as fun and uplifting to the adult pain*HEALTH* website which had a more monochrome colour pallet and described as dull and less engaging.	*The second one was nice because there was this young-looking website for the target audience, who I believe is youth. So, it was colourful and I liked how there were pictures of relevant cases to the content, so the pictures were relevant and also it was very tidy as well, it was clustered well…(P7)*<br><br>*It was nice bright colours as well, which I think was quite appealing. They were appealing in the sense that they were bright and colourful, which I like, the bright and colourful colours. It's more attention-grabbing than, I don't know, dull, dark colours (P8)*<br><br>*I felt like the colours were good. I felt like the design was youth-focused, but not necessarily exclusively youth-focused, if you know what I mean? If a 50-something year old were to look at it, they wouldn't just be like, "Oh, this is dodgy". (P11)*<br><br>*. I really thought the website was just so well-targeted to the young adult audience and the adult version was very black and white and a bit of red through it. Young adult websites are very much colour and more engaging and fun. (P9)*
Subtheme 1.1.2: Vibrant colours associated with eliciting positive emotions<br>Using vibrant colours in digital tools also appealed to participants as it was noted that seeing bright colours was uplifting and associated with positive thoughts and emotions rather than sad or negative thoughts.	*Again, it's very bright, like the painHEALTH website, so it does make you feel a bit better going into it because of that (P1)*<br><br>*I liked the blue as well, that's a nice calming colour. (P6)*<br><br>*I really enjoyed the bright colour scheme with the blues and the purple and the pinks. I thought it was really calming and really interesting, as well, for a young audience (P9)*<br><br>*I actually preferred the pink and purple to the blue and black just because I think the blue, to me, it's more a representation of sadness than anything else. So I think,*

<table>
<tr><td></td><td>for me, if you're going onto a painHEALTH website it doesn't really - I wouldn't really want to see it in blue. I just feel like it would maybe make me feel a bit worse looking at it in that context. (P1)

So the colours, the pictures, it was more interactive for teens especially. It just seemed a lot more fun to interact and make your way around and it wasn't as serious, like the outlook on it with all the colours made it less serious, so it wasn't, "Oh pain, oh!" It wasn't all sad and gloomy, (P13)</td></tr>
<tr><td>Subtheme 1.1.3: Different shapes, curved edges, lots of images and illustrations are modern design features that appeal to users
Young end users were unequivocal in their preference for digital interfaces with modern designs that were engaging and playful which motivated them to engage with them. Modern design features included use of non-linear shapes, visual images (illustrations and pictures) and call out text.</td><td>I really enjoyed how the layout was all the bubbles instead of being just straight text or in boxes, it seemed really interesting and fun. (P9)

The first thing that I noticed was actually the illustration and the imagery that came along with it; I thought it was quite catchy. It's the first thing you see when you look at the website and I think if it's up-to-date and relevant to today's society, this society is really technology-based. So by looking at that, it was really attractive. If someone was to look at that, they're more inclined to go back and use it and want to look further into it. So I thought that was a good thing, firstly (P15)

The layout was more casual feel and wasn't as formal and was something that the government would've - it wasn't as much like that. The colours were really helpful, there was bright yellow and there were all these different colours around, and the pictures were really helpful. (P13)

Even things just like soft edges of text boxes rather than hard lines/more clinical-sided boxes were very much more visually friendly… I felt it was almost a playful interface as far as the colours and the shapes, rather than the second one was that more, kind of, clinical, I felt, visual structure that was less fun and interactive… It was just the colour of that first one and the shapes that made it look more appealing (P2)</td></tr>
<tr><td colspan="2">Key theme 1.2: Present content in ways to optimise reading and comprehension
The need to present information using different ways to optimise reading and comprehension was predominantly discussed in relation to the painHEALTH website prototypes. Participants suggested four main ways to improve acceptability of the prototypes, where acceptability related mainly to the information being relevant to users:
i. Use of different formats to deliver information (e.g. video, audio, text, illustrations).
ii. Careful attention to font – the size, type and colour all impact readability (particularly if user feeling unwell/fatigue).</td></tr>
</table>

iii. Using positive, non-jargon/technical language and less formal wording to enhance engagement.<br>iv. Short and concise information with links to additional information/external resources.<br><br>Illustrative quotes relating to the four subthemes are outlined below.	
Subtheme 1.2.1 Use of different formats to deliver information (e.g. video, audio, text, illustrations)	*I found it all very easy to read. It was nice big font with a good balance between images to break up the text, as well as videos and stuff like that as well. (P3)*<br><br>*Generally the amount of text in there, I felt like all of them were a one to two minute read, kind of thing, with a short video in there that would just decrease the amount of text but still get the message across, which I thought was really good. So I think, yeah, the videos were all really good additions because instead of being three paragraphs to explain this thing, it can just be said in one short video. (P3)*<br><br>*You have different pictures and videos on there as well, which is nice. If you weren't really feeling like you wanted to read a lot of the information, it's nice that they have the video option there as well for a lot of the stuff. (P1)*<br><br>*I think it was good to have the videos and the person going through their story at the top, so you could have the option to listen to him instead of reading through (P6)*<br><br>*I really did like the integrated design of it. I felt like it was very eye-catching and the different multimedia presentations, the video, the text and having lists and such was something that I found really helpful (P11)*<br><br>*I felt like there needs to be an audio version as opposed to just the video and the text. I feel that would be good for accessibility and especially if people's eyes hurt and it's getting to the end of the day etc., that would be something that would be at least an option that would be very useful. So that lack of option was something I didn't like. (p11)*<br><br>*I quite liked having all the photos and stuff there, like the photos of the patients rather than having long lists of - because I'm not sure if it was specifically for the people's stories, but one of them just had a list of options, the other one had the photos and you'd scroll down the different photos with the people's names. So having that visual side of things was really good (P3)*

Subtheme 1.2.2*. *Careful attention to font – the size, type and colour all impact readability (particularly if user feeling unwell/fatigue).	*But generally both of them had relatively large font, but not so large that you have to be scrolling down for ages to actually finish the story. The rounded off font of the colourful one was quite nice, like easy to read. (P3)*<br><br>*But the writing itself is a decent size, so for anyone that does have a bit more trouble with that, like if they do have a headache, the fact that it's a bit bigger is better as well. (P1)*<br><br>*I guess you could make it bigger text, because it'd be easier to see for younger people (P4)*<br><br>*I think the font was one of the first ones to come out. When you look at the font, it's readable and when you compared it with the old website it was easier to read, I think, because of how the font characters are shaped or the letters, like circular or edgy. So if it's edgy it doesn't look very nice and it gives a bit of stress when you're reading it, so I think circular is good (P7)*<br><br>*Some of the text colour is very light grey/blue on white and I found that less easy to read than the dark grey text, to the point where I found myself not reading the headings of things. I just sort of skipped past them… The dark green/grey of the links looks good and the black, it's like a dark grey on white, that works well as well. The menu colours with the white text on blue is also easy to read, but I'm now noticing that when you hover over the menu then the text turns to white text on that same light greyish/blue and it's completely illegible, you cannot read it. (P10)*<br><br>*I think the colours are quite good as well, they're a nice blue colour so it's easy to read, it's good on the eyes. The font size was good also (P8)*
Subtheme 1.2.3*. *Using positive, non-jargon/technical language and less formal wording to enhance engagement.	*but all the information was in casual language and not all scientific and scary that you read two sentences of and don't want to read anymore of. So it was all understandable and it could've probably had some more on it… I think there was some like this disease, what to do, kind of thing, like all the information-type ones. (P13)*<br>*I guess the thing that struck me the most, I guess, in a good way was how it was sort of almost unprofessional, like it was just asking these questions with almost like childish cartoony images. Even though I know from a physio student perspective that the questions that were being asked are really important and*

	there's a specific reason why each of those questions, sleep and physical activity and stuff, are all being asked, it didn't come across like that, which I think is a good thing. It almost disguised these really important questions into just like, "Oh, how are you feeling today? How is your mood?" So it seemed nice and laidback in that sense, which was good because it came across as less of a tool for professionals but more of a very easily usable and understandable way to keep track of my pain. (P3)<br><br>*I mentioned previously, I found that the framing of the tone of the titles of the articles in the prototypes were much more positively framed than the original one, which I really liked. I can't remember an example right now, but I remember commenting that I liked that positive spin. I think that is encouraging and makes people more interested to read the article.(P2)*<br><br>*It's not "for kids", there's no being spoken down to at any point with this site. (P10)*
Subtheme 1.2.4 Short and concise information with links to additional information/external resources.	*Yeah, if there was any further links to go to, websites, but they're all quite small and easy reads to go through. I think they were quite appropriate for what you want to be doing. (P6)*<br><br>*I feel like also linking external resources would be a good thing and maybe other pain apps etc. Even though these are direct competitors, the fact is that when you are chronically ill or in a lot of pain, like I am every day, you want as many resources as you possibly can so that you can filter out through (P11)*<br><br>*The shortness of them, the conciseness is pretty helpful as well, so I can just sit down for five minutes, read it and then take it on, see if it works and if it doesn't work, find another one, like, find a method to suit me better. (P14)*

<table>
<tr><td colspan="2">META theme 2: WEBSITE CO-DESIGN PROMOTING USER ACCEPTABILITY AND ENGAGEMENT</td></tr>
<tr><td colspan="2">Key theme 2.1: Acceptability of content foci
Participants noted that the painHEALTH website provided important information that addressed the needs of users. However, participants also highlighted further improvements to optimise the acceptability of content including the need to break up large chunks of text with images, diagrams and call out features as well as wanting more information on available pain management services and more specific directions on how to access help.
These subthemes are discussed in further detail below.</td></tr>
<tr><td colspan="2">Subtheme 2.1.1: Information matches user needs
There were three key features of painHEALTH website identified by participants that were important in addressing the information needs of users:
(i) Good breath of content on practical day-to-day management.
(ii) Personal stories are relatable and a standout feature in reducing isolation.
(iii) Self-checks empower users in taking control of self-management of pain.</td></tr>
<tr><td>(i) Good breadth of content on practical day-to-day management
The breadth of content in relation to practical day-to-day management of musculoskeletal pain was appreciated by participants as it was reported by a few that this information was often hard to find.</td><td>It’s got plenty of information on different types of pain, things like sleep, which is really helpful as well ‘cos that’s a big part of it, and also information on who you could speak to if you’re having further issues, which is really nice as well because there’s not a lot of that information elsewhere (P1)

Other than that, I liked that it was relevant, like management of pain and there was a bit of information about diet I believe, which I thought was something that’s usually overlooked and very important in terms of inflammation and chronic pain. (P7)

The main thing that I always look for in these painHEALTH sites is information about drugs and addiction, which this site has. So it ticks everything that I really want to know about. (P10)</td></tr>
<tr><td>(ii) Personal stories are relatable and a standout feature in reducing isolation
Participants liked the use of real world stories on the websites as it resonated with their individual experiences of pain, reduced feelings of isolation and showed how others had learnt to cope with their pain.</td><td>There were good stories and that also gives a personal touch about what those people were thinking going through that and that makes you think okay, you’re not alone in this situation, there are others in the same shoes, which was a good aspect (P7)

Also, I found that the stories themselves are pretty handy… I think it shows me that I’m not the only one doing it, but they’re also demonstrated methods of coping with it within their everyday life. That was the key bit. (P14)</td></tr>
</table>

(iii) Self-checks empower users in taking control of self-management of pain.<br>A couple of participants noted that the self-assessment tools were also helpful in assisting them to evaluate their current pain status and then seek relevant assistance from health professionals to improve pain management.	*I also liked how there was a self-assessment tool which helped with evaluating yourself, because sometimes you get by with what you have, a similar situation with pain or diet or whatever you're going through, you take it for granted, forget about it for a while. It's there, but it becomes your daily life, but when you start something like a self-assessment tool that makes you realise that that's how it was before, then you can improve and you can become better, you don't have to just get by with that pain that you have. It was a bit eye-opening, so I liked that part of it (P7)*<br><br>*I think what I liked best about the website is that it's an easy method of just approximating, like, do I have a risk for musculoskeletal - the testing, the tests are the key things… I found it helpful that the self-checks can also then be deferred on to a medical professional and they can also then interpret those results as well, rather than sitting down with your health professional and going through similar questions to get a similar result. I think that was handy. (P14)*
Subtheme 2.1.2: Information pages too text heavy and need to be broken up with use of images, diagrams and call out text features<br>Older participants (23-24 years) were particularly critical of the heavy text focus of the pain*HEALTH* website and strongly advocated for improvements including chunks of text to be broken up by using 'call outs' and greater use of images/interactive features (such as video content). Furthermore, it was suggested that the most important information should be featured at the top of the page supported by further detail below.	I *felt further work could be done to improve. Things like having a lot more figures and images in the text, so like the articles or explanations, having a lot more diagrams that are explaining some of the science (P2) [painHEALTH]*<br><br>*Yeah, definitely felt it was way too text-heavy. I'm an older, more science literate/health literate individual and I just thought people will be zoning out quite a lot. There were some really good summary points at the bottom of each of the articles and I almost felt like they should've been at the top of the article, like key takeaway points and then use the extra detail if you want to read further. Yeah, the articles are too long and I felt that people would flick out of that page before they even got to that really valuable information at the bottom. (P2)*<br><br>*I think some extra headings in some of the articles could've helped break it up, but potentially even breaking articles up into smaller articles could've been beneficial (P2)*<br><br>*In this day and age, it's probably more text than the average person is used to, but I found the font and size pretty good... having an infographic, that might be more aligned with what people are used to now scrolling through Instagram where it's like two sentences and a picture, two sentences and a picture, two sentences and a picture; it's so narrowed down (P5)*

	Maybe have the major points outlined at the top, for example (P6)<br><br>*I think for the layout of some of the articles, I remember just saying that they could've been probably a little less text with more of the images to keep it engaging. Some of them got a bit long. (P12)*<br><br>*I found that the quotations in the stories got a bit repetitive, especially when there were big, bold quotations and then in the next bit of text it was the same thing you were reading again. I found that a little bit frustrating. (P9)*<br><br>*What could be more helpful is if certain topics etc. are bolded, like certain words within the website itself, just so if people are scouting through looking for particular key words. Especially when you have less resources to really look and talk about to observe things and such you're going to want to find things quickly and efficiently…For example, "arthritis", like particular conditions and, say something that's like "what you can do to live". Like, maybe more efficient phrasing, like "lifestyle tips" or something like that, and maybe have headlines bolded etc, just for the sake of convenience of information (P11)*<br><br>*I felt like there was too much jargon, especially for a youth audience. On that, if you are having a wide range of youth viewing that website, potentially having different levels of engagement with that information. For example, like an article explaining what is pain or the science behind pain: are you working to the lowest common denominator? If that's the case, are you pitching this to eight year olds as well? Just making that information accessible and maybe that is having sections that are accessible to different levels of knowledge or different levels of literacy as well. (P2)*
Subtheme 2.1.3: More information wanted on services available and how to access help<br>Several participants wanted to see more specific information on the types of pain management services available, the range of health professionals involved in these services and how the services could potentially help them. Additionally, it was highlighted by a few older participants (23-24 years) that it was difficult to know how to access health services so clearly identifying the steps would be beneficial.	*My next recommendation which I had was about the contact page of the website. The contact page of the website just had listed a whole heap of hospitals and health centres. I'm not from Perth, so I looked at that page and thought, "Well, I don't know what type of pain services they offer. Do they have an outpatients' clinic? Do they have pain counsellors? Is it more like a mental health benefit?" (P2)*<br><br>*also in terms of the support link, like the contact page, I just suggested to* [research team member] *that because that did seem a little bit list-like, I think it was for both websites, to include maybe a little Google Maps image in there of where the*

	hospital or whatever is located. Again, just to break it up visually so there's not such a - sort of just seems like a random list of hospitals and you don't really know where to start, whereas if it's broken up a bit more you can. (P3)<br><br>*When I was doing the interview, I mentioned to* [research team member] *that I think the Help section, where it gave the list of the hospitals and healthcare centres, that needs to be a bit more refined so that it's easier to see what you were looking for. I'd like to see a website, like a hospital website and then further contact details on that website or instructions on how to get to the details on the service and what they can do [P6]*<br><br>*there was a Contact Us page and there was about other referrals or other support. There was information about hospitals, so I was a bit critical about how individual hospitals are going to benefit people in the community. For example, I thought it would help if there was information about a certain department that would be beneficial, maybe a pain management department or something like that, information about their website or their contact details, if they are doing any community work. Otherwise, if there is any information about the website of a hospital, if you go to a website there's so much stuff there you get lost. If you go to the switchboard, if you call over the phone and you ask questions, they're going to ask if you're a patient or not. So with that referral or support documentation or other avenues that you can go to, I think it should be relevant and specific. (P7)*<br><br>*At the time I was also commenting on there were personal stories, so there was one in particular that they showed which was a teenage boy that basically, long story short, got help with managing his pain through yoga, meditation, massage and physio, a whole range of things. I thought that story was really great, but I felt what that story was missing was how he reached out for help. So it talked about that he'd had a situation at work and that he needed to reach out for help and then he found all of these great things, but it didn't talk about that process and I feel like, especially with adolescent boys, there's a bit of a stigma about reaching out for help, especially in the pain space. So, I feel like that story could've been - like it was a short story. I felt that the length of that story was really good, but just that that link of "and he reached out to this service" or "he googled the painHEALTH website". What was it? Did he ask a friend? Just having those little prompts I think, of how people seek help would be really valuable… So just knowing what services*

	are actually available or is it just like a pain clinic. I felt that that could be really linked into those personal stories, linking why would you go to each of these different services and how could they help you (P2)<br><br>*The last thing was they had a quiz scoring about disability from pain. I may have got that wording wrong, but basically like a quality of life kind of scoring which, I don't know, I felt a little bit uncomfortable about that actually because it kind of just came out with a score saying you've got a lot of probability of your life being long term affected by pain . Yeah, I can't remember the exact wording, but if someone came out with a bad score, what psychology, what social services are there when they're doing this online score to help them through that? Where do they go from there? You had options to download or print this score, but then what do you do with that? Are you going to take that to a GP or a psychologist and what are they going to know about that score, but also what impact could that have on the individual after doing that I think was my concern…I also think if that kind of information is going to be on there then there needs to be some sort of Lifeline link on that page as well. When that comes up I just thought that was a little bit risky but yeah, just always linking it back to how do they reach out, where do they reach out? If they say things like, "Okay, I could benefit from help" I think that's a really crucial - if they've got to that stage of change mentality then great, you want to provide the easiest path to them actually getting help. (P2)*
**Key theme 2.2 Optimizing navigation**<br>The majority of participants found the pain*HEALTH* prototypes easy to navigate. Features that facilitated easy engagement included a drop-down menu, clear website layout and simple design. In terms of improvement to optimise navigation, the use of a search bar and minimal clicks to get to relevant content were suggested.	
Subtheme 2.2.1 Drop down menu feature widely liked and assisted easy navigation<br>The drop-down menu feature in one of the pain*HEALTH* prototypes was widely praised by participants as facilitating quick and easy access to all sections of the website. It also allowed users to see "at a glance" the range of information available and easily navigate to the relevant sections.	*Yeah, the banner at the top that would have the links to stories and contacts to find support and stuff like that. So when you'd scroll down that would still appear at the top, whereas one of the other websites [painHEALTH] you'd have to scroll all the way back up, well, you know, back up to the top to be able to actually click onto that banner onto the different links. So that was something, it was more accessible to have that pop up as you scrolled down without having to scroll back to the top (P3)* [relates to PROTO 1v1 and PROTO 2 v1]<br><br>*Everything is easy to find because it's labelled very nicely at the top there for you and it's all dropdown, so it's really easy to go into what you're looking for. (P1)* [relates to PROTO 1v1 and PROTO 2 v1]

	I quite liked how it was quite easy to follow. The menu bar at the top, it was quite easy to understand where you're going to. (P6) [relates to PROTO 1 v2]<br><br>*The tabs are very good, like when you go to a tab it drops down without you clicking it, which is good, then you can like navigate yourself well. I was going through on my PC so it was nice, (P7)* [relates to PROTO 1 v2]<br><br>*I really liked the ease of access and how everything was really at your fingertips. If you just clicked on a heading it would give a breakdown of the links, so that made movement around the website very easy. It was all very self-explanatory with the headings up the top because it had the main headings, which you could click onto. But as you hovered over them it would come up with the subcategories for them, which I think really, it just made navigation so much easier. You could see at a glance what you were looking at, rather than having to click between different sections. (P8)* [relates to PROTO 1 v2]<br><br>*Probably that it was easy to use. Everything was easy to find and the diagrams and dropdowns were really good (P12)* [relates to PROTO 1 v3]<br><br>*Also, I liked how easy it was to find things, so how you've got the ribbon up the top that's got little categorised sections and then within there you've got other things. I liked that, I found it quite easy to work around. (P15)* [relates to PROTO 1 v3]
Subtheme 2.2.2 Website layout design clear and easy to use<br>Participants noted that the simple layout design made the website prototypes easy to navigate, particularly the colour coding of different sections.	*I felt like that in terms of the number of headings and stuff it was good because it sort of started relatively broad, say, in stories and then you could narrow that down further, or screening questions and then narrow that down further, "Okay, there's these different screening questions, I'm looking for the Orebro" and I found that pretty easy. If you had too many more headings at the top I feel like you could get a bit lost and not really know where to start, but it was something like five or six I think, from memory, which was a pretty good amount, something I'd probably normally expect from a website. It just meant that you could click onto that link and then look further, so you might click on the screening one and there's three different options that you can choose from and it was pretty clear as to how to navigate through those and which one you would use and stuff like that.(P3)*

	it was clustered well, so it was easy to navigate… the home page was well done, so you knew you were on the home page and then you could go wherever you wanted to head towards. (P7)<br><br>*It's very clearly laid out and really easy to navigate without being, I don't know, patronising. I've been on patronising information sites before. (P10)*<br><br>*I also really enjoyed how each of the sections of the website were all coloured coded together, like all of the stories were in purple and all of the management information was in pink. (P9)*
Subtheme 2.2.3 Search bar considered an important feature which would assist looking for specific information<br>Several participants highlighted that the addition of a search bar would enhance navigation of the website prototypes. This was discussed in the context of being helpful if a user was looking to find specific information quickly or having trouble finding the information they were looking for.	*I think maybe if there was more information on there they would probably need to add in some sort of search bar but otherwise, at the moment, the way it's setup it doesn't really need one, from what I could see… I think it would probably be helpful. Just for anyone that does have a bit more trouble, sort of, technology-wise it might be beneficial to have on there. (P1)*<br><br>*The search bar would make it easier, but it was pretty straightforward and I was able to find what I was looking for (P4)*<br><br>*Was there a search button? I think that would be really helpful, if you could type in your keyword and then it brings you suggestions to what page or what tab you need to click on. (P6)*<br><br>*It was difficult when you were specifically asked to look for something. So I was asked to look for Max's stories or something and it was hard to navigate where to actually find these ones, but if you weren't looking for something specific, like Max's stories, then a normal, everyday user would have found it perfectly fine… I think that* [a search bar] *would really help matters (P13)*<br><br>*but often I think a search bar is quite handy. I think it's necessary for someone, even first time users to a website, if they want to find something quickly. (P15)*
Subtheme 2.2.4 Preference for minimal clicks to access information<br>A couple of participants strongly articulated a preference for minimal clicks to access information. Participants wanted to be able to move across different sections easily without having to return to the home page or have	*So I found it easier if it was just less clicks to have to get to actually what you wanted to get to. Also with that it meant that another one of the things with the websites was that once you finished the Orebro you had to click back, there was a little arrow back that said "back to the home page"* [PROTOTYPE 1 v1 and PROTOTYPE 2 v1] *rather than another one always had the banner at the top as well*

a middle page between the menu bar and the information the user wanted to access.	*and it meant you didn't have to click back to home page, you could just go straight to the next thing that you wanted to go to, whether it was the stories or whatever [painHEALTH]. Which I found it was better to not have to go and click back to a totally separate thing because I felt like that was the only way I could go and it's just more clicks away, which in reality is only half a second difference of your time, but in terms of ease of use I found, yeah… generally, as long as there's not so much stuff on one page, I think the less clicks the better (P3)*<br><br>*probably an update to the home page so when you click on the menu bar you don't go to another page that's got all the links there, you actually just go directly to the page that you're looking for from the menu bar, rather than going from the menu bar to another page to your page… I think the middle page between the menu page and the actual page I wanted to go to was clunky… I think if you can reduce as much, as I said before, I think the middle page should be cut out completely and just a more modern menu bar should point you directly to the page in the right direction for accessibility and navigation. (p14)*
**Key theme 2.3 Website supports self-management**	
Subtheme 2.3.1 painHEALTH perceived as a valuable information resource that contains practical tips, helpful contacts and insights into specific conditions<br>Participants perceived pain*HEALTH* to be a valuable resource, particularly in relation to providing practical and detailed information about day-to-day self-management e.g. pacing, managing stress. The personal stories resonated with users and made them feel less alone and the list of contacts to seek further assistance was also considered highly useful.	*I think if I had other people, like friends or family, I would probably refer them to the website as well…I think it is good in the sense that it does cover other aspects and it links other health conditions and does talk about having stress, what that can do and how that has a knock-on effect, exercise and things like that. So I think that's good, how it does give you this other information as well. (P9)*<br><br>*it's amazing to think that things like this are coming out because when you first get diagnosed or whatever there really aren't places like this. So it's exciting to see that people are starting to think about this. It's great. (P12)*<br><br>*I think the website is quite good for further information and reading other people's stories and relating to it and all of those types of things, and as well how if you want any further information it's giving you all the people you can contact and stuff as well. So I think that was really informative. (P15)*

<table>
<tr><th colspan="2">META theme 3: APP FUNCTIONALITY SUPPORTING PAIN SELF-MANAGEMENT</th></tr>
<tr><td colspan="2">Key theme 3.1: iCanCope app supports self-management through several features
Several features of the iCanCope app were identified by participants to support self-management namely:
(i) Daily check in – which asks users to rate their pain, mood, sleep and physical activity each day using prescribed scales.
(ii) Pain tracking log - based on daily check in information over periods of time.
(iii) Setting goals – a separate section where users are asked to set goals and monitor progress towards achieving goals.
These are discussed in further detail below.</td></tr>
<tr><td>Subtheme 3.1.1: Daily check-in encourages self-reflection and identification of broader factors that may impact on pain
All participants nominated the daily check in as the feature they best liked as part of using the app. By rating their pain, mood, sleep and physical activity each day, it was highlighted that this encouraged them to reflect on their pain and made them aware how broader factors such as mood, sleep and physical activity could impact on their pain, both positively and negatively. For several participants, this was the first time they had learnt about this. Self-reflection also made participants think more about how their daily activities were affecting their self-management.</td><td>So I think it was good for bringing to light some of those things, because normally you wouldn’t typically finish your day and be like, “I was in a lot of pain today. Why was I in a lot of pain?” and this helps to start those, I suppose. (P3)

I think it is good because, for me, I wouldn’t personally normally think about how everything is affecting me. And it’s asking you not only to say what your pain level is for the day, but also how it’s affected you as well, so you’ve been able to do less or do more or you have a better mood than you would normally or your energy level is better than normal. Because I might feel that I’m in pain all the time, but I don’t normally think about how it’s affecting everything else, so I think it is good that it makes you aware of all of that as well. (P1)

I thought it was very helpful to see how my pain has changed over time and how the other stuff has contributed to it… I thought it was very helpful to see how it can affect your pain or how your pain can affect your physical activity and the information was very useful… It was very helpful because it teaches you how sleep can be important to managing pain or how pain can influence it. Yeah, I hadn’t really heard about it before (P4)

…and it gives you a bit of self-reflection. Then you can start to realise that there is a pattern here. You know, if my sleep wasn’t good then maybe there’s a correlation with the pain or how I’m feeling also maybe affects the pain. It might be a loophole where maybe pain affects my mood, but usually the pain is always there, so my mood changes depending on the circumstantial stuff going on during the day. It’s also a good journal as well. You ask yourself how was I today, so it was a good journal for self-assessment and realising your patterns (P7)</td></tr>
</table>

	I thought it was really good to have a check in every day so you could see alright, I wasn't feeling too good on this day, what did I do that day that made me feel like that? And I really liked how you could look at the trends of what your pain was and how the app would say if you were going through a rough patch or if you're doing really well, I liked that. (P9)<br><br>*So it's really good to have that and recognise that could be causing the pain, so you can look back at that and see if I had pain that day and it will make the connection between the two so that really helps…Yeah, for the patient, like even my parents were looking at it and going, "Oh, that's awesome". Even them being able to track that and how I'm feeling at the end of the day, that was really, really helpful (P13)*<br><br>*I think the app was something I could open and start tracking pain levels and use for correlation of particular dates or using just the 1 to 10 scale. I think that was really handy… Yeah, in terms of tracking, logging pain, I think that was crucial. The fact that it starts looking for rough patches and overall performance, I think that was helpful. It allows you if you go to the history to see via colour gradients your pain, emotions, energy levels, physical activity, and start making some correlations to what you did that day and your mood to your actual pain level. I think that's a good overall feature about the app. (P14)*<br><br>*I liked how it covered a range of different areas, including mood, just because it first of all helped you recognise a pattern or not a pattern. So there were a few days where I had a lot of pain, but I was sleeping really well and I was exercising really well, so I was kind of like, "Okay, maybe it's my workstation. Maybe it's just because I am sitting too many hours and if I'm exercising at the end of the day, that's just not going to be enough". I just moved to a new country so my stress is going to be higher for a while, so then having that mood pickup was kind of nice for me to feel like, "Is my stress coming down? Is my pain coming down with it?" Yeah.* [P5]<br><br>*Yes. I think it was good actually having something to help you take note of how you were today and what may have been contributing to the way that you felt. So if I had maybe worse pain on one day and then I noticed actually I had low mood, low sleep and low physical activity then I'm like, "Oh, okay. Maybe tomorrow I can try*

	and do a little bit more physical activity, try and get a better sleep, and maybe my mood will be better and my pain will be better" sort of thing (P3)
Subtheme 3.1.2 Pain tracking supports positive pain care behaviours (monitoring, self-reflection, goal setting, coping, progress over time) Participants also valued being able to track their pain levels over time and actively using that data to change their self-management behaviour. For example, trying to increase their physical activity or actively lift their mood as well as assisting them to pace their activities appropriately depending on their pain levels.	*It was interesting to see how much sleep I got related to my pain and my mood etc… It's like, maybe I should sleep more (P6)*<br><br>*Yes. I think it was good actually having something to help you take note of how you were today and what may have been contributing to the way that you felt. So if I had maybe worse pain on one day and then I noticed actually I had low mood, low sleep and low physical activity then I'm like, "Oh, okay. Maybe tomorrow I can try and do a little bit more physical activity, try and get a better sleep, and maybe my mood will be better and my pain will be better" sort of thing (P3)*<br><br>*Yeah, I think so, because you might not be that conscious about how your mood is for the day and when you are made more aware of it it's easier to try and uplift your mood for the day if you're not doing so well, try and stay more positive, which is good. (P1)*<br><br>*So obviously with pain it's hard to always be physically active. So it is a bit confronting sometimes if you're looking at it and you're going, "Oh well, I haven't actually been very active today". I think it can make you feel a bit worse realising that but, at the same time, it could also push people to try and do more as well…I think it's good that they do have the articles and everything on there as well to try and help you figure out what sort of exercise would be better for you with chronic pain, because that's a big thing too, not knowing what you should do that would actually not be as heavy impact on your body (P1)*<br><br>*I liked how it covered a range of different areas, including mood, just because it first of all helped you recognise a pattern or not a pattern. So there were a few days where I had a lot of pain, but I was sleeping really well and I was exercising really well, so I was kind of like, "Okay, maybe it's my workstation. Maybe it's just because I am sitting too many hours and if I'm exercising at the end of the day, that's just not going to be enough". I just moved to a new country so my stress is going to be higher for a while, so then having that mood pickup was kind of nice for me to feel like, "Is my stress coming down? Is my pain coming down with it?" Yeah. [P5]*

	Yeah, I would say it did and it did remind me that it's okay if you're feeling a bit more pain on one day and then take a little bit of a step back the next day. (P9)
Subtheme 3.1.3 Encourages setting of goals and monitoring progress<br>The goal setting feature on the app was also deemed highly useful by participants. Many reported that they had never thought of setting goals prior to using the app and had found the suggested goals provided by the app as extremely helpful in identifying which self-management areas they could do better in. Setting goals also motivated users to actively engage in self-management strategies in order to achieve their goals.	*In terms of goal-setting, I guess I always have goals in the back of my mind and I'm always thinking to myself, "Okay, how many times have you actually exercised this week" and this and that. (P3)*<br><br>*I think it's a good idea having it because you might just be down in the dumps, you don't really think you can do anything to improve, but setting basic goals and the fact that they suggest some goals as well, depending on how you're going with your check-ins, can be really helpful . 'Cos even just trying to improve how much sleep you're getting in a night or how much activity you are doing during the day can be really helpful. (P1)*<br><br>*I really liked the suggested goals; that was really cool. It made me realise areas that I might be doing well in as opposed to areas I might not be doing as well in. In terms of social goals, I personally tend to work pretty hard to meet my social goals anyway. I do a number of activities, so when I looked at the suggested social goals it's like, "Okay, yeah, I think I have a handle on that" but then if you look at the other ones it's like, "Okay, I probably could use some help setting specific goals through this app". So yeah, I did really like that (P5)*<br><br>*But one thing that's really good about it is that it has this rotating wheel of suggested goals, which are great. When I flick through them I think, "Oh, yeah, that's a really good idea actually. Yeah, I could be doing this". (P10)*<br><br>*I filled out the goals and I thought the notification settings were also really good for that as well, because that reminds you not only to check in but to do the goals as well, so that you have a space to be able to remember to practise whatever you'd written in your goals. I thought that was great. (P12)*<br><br>*That was awesome. That part was really good because you can track personally what your goals are, when you've completed them, whether they're short term goals or long term goals. It could be, "I want to be a teacher when I grow up" or it could be, "I want to get out of bed today". It didn't put a number or level on it, so you could achieve it. It was really open, which was really nice. (P13)*

<table>
<tr><td></td><td>I think that section of the app was pretty good and I think the fact that you can then set a time period for you to complete that set goal, making it more a SMART goal, I think that was pretty helpful. I think one of my goals during the trial was to do at least 30 minutes of exercise every day. I managed to do that every day through the goal setting. (P14)</td></tr>
<tr><td colspan="2">Theme 3.2 Utilise pain tracking information to inform and support clinical consultations</td></tr>
<tr><td>Whilst a minor theme to emerge, a couple of participants noted that the pain tracking information provided by the app would also be helpful in informing discussions with health professionals to show them progress or identify noticeable trends in relation to pain self-management.</td><td>I think it could be used for both, like for just monitoring it yourself. If health professionals need to see how it’s changing, that would be a good tool for them to use. (P4)

Yeah, like people with undiagnosed illnesses and pains in their body, they can go and be like, “Yes, okay, this proves that I have been in pain. I can go and show my doctor this and show them the trends” kind of thing. That would be good. Make it relevant to GPs where they can look and see, “Oh okay, they have been experiencing pain and not making it up”. (P6)</td></tr>
<tr><td colspan="2">Theme 3.3 Usability
Usability refers to the interactive user experience with the iCanCope app. Whilst participants enjoyed several interactive features of the app, in particular the engaging and easy to use daily check in function, they also had a considerable number of suggestions to improve the usability of the app for the target audience of users aged 16 to 24. These are discussed in further detail below.</td></tr>
<tr><td colspan="2">Subtheme 3.3.1 Daily check in feature engaging and easy to use
Participants consistently reported that the daily check in contained interactive features that engaged them over time. There were four main components identified which assisted this interaction, namely:
(i) Easy and efficient to use; low burden.
(ii) Scales intuitive and relatable.
(iii) Reminder to check-in.
(iv) Visual diagram of mapping pain on body and using words to describe pain.</td></tr>
<tr><td>(i) Easy and efficient to use; low burden
Participants reported that the daily check in was quick and easy to use, taking only a few minutes to complete each day. This was an important component in keeping users engaged over time as it wasn’t considered a burden to log information about their pain on a daily basis.</td><td>So [daily check-in] was really straightforward I found, like scrolling through the different levels of pain and then going through activity and mood and stuff for the day. Again, just one of those main bars down the side, main options down the side, which is very easily accessible and it only took a minute or two, which I thought was really good. (P3)

It was very easy to do the daily check-ups and put in your pain levels and mood stuff (P4)</td></tr>
</table>

	I just like how easy it is. It doesn't take very long to check-in and it's very easy to see how it's changing and what's impacting it (P4)<br><br>*I liked how it was a really quick and easy to check in, like you just clicked on, pressed "check in" and it was five or six simple questions. You could check in and there were easy scales, it was fun to do with the little pictures. (P6)*<br><br>*The check in is very easy, it just takes 15 seconds to complete, so I just go on it and do my check in and, at the same time, you check in for your pain, how you're feeling, how you slept… It was short and you knew that if you went to do your check in, yeah, it'll take you a small amount of time and you're happy to do it. If I knew that it was going to take me five minutes I would probably stop bothering after a while because that's a continuous effort. (P7)*<br><br>*All the questions are really good, self-explanatory, and I felt like there were enough questions to make a good judgement, whereas other apps that I've seen have just been two questions, not necessarily everything. I think it asks about your pain, your sleep, your physical activity, energy, mood, everything, so it covers a good basis, while also not taking half an hour to fill out. (P12)*<br><br>*It still allowed you to ask all the necessary questions but without all the nitty-gritty stuff, like what was your pain, how did you do today to deal with this or things like that. It was really, really quick and easy to do (P13)*<br><br>*That was a fairly easy process. I had a notification, go to my notification, so I'll click on that and that'll take me to the app and then I'll be asked to do a basic check in. Then it'll go through your - it's got the dial for the 0 to 10 and representations of each one. Yeah, that was easy to do. (P14)*<br><br>*Well, definitely I found it easy because it wasn't like you have to click into one answer, click into another answer; it kind of was like the answerer and then it takes you to the next section automatically. So yeah, it was quite helpful (P15)*
(ii) Majority of users found scales intuitive and relatable<br>Scales used to rate pain, mood, sleep and physical activity as part of the daily check in were also perceived as easy to use by the majority of participants. Many liked the simplification of scales which used one word	*I actually really liked the sliding bars, the rotating bar thing for zero to five or zero to ten, whatever, in terms of the amount of pain and those descriptions with an image to assist in being like, "Yeah, I just felt alright today in terms of my mood" or whatever. And I think also the descriptions, so for mood, for example, it wasn't "Felt*

descriptors or a cartoon character and found that it was easy to relate in terms of how users were feeling at the time.	*really happy and positive/felt a little bit happy and positive/felt pretty average" it was actually just simple terms like "Great/good/meh" and "bad/worst" and I think the simplification of those words was really good because it was like yeah, "meh" is actually a pretty good way to explain how I was feeling… I thought it would be very applicable to many people my age who don't have a health sciency background as well. (P3)*<br><br>*So yeah, for the mood, for the sleep, for the physical activity, I think it was all, again, explained with nice simple terms and just that numerical grading scale with zero to ten for the pain and for the other things using the words, like, "meh/good/great" I think was really good. (P3)*<br><br>*I really liked how the icons were so easy to read. I think when you're in pain, sometimes it's very hard to rate yourself onto it, but with the little icons it was very helpful because I'd be like, "Oh, I'm not really doing that face, but I'm feeling like the other one". (P9)*<br><br>*Once again, I love the icons. They're so good at really detailing what the question's asking you rather than just a scale of numbers; I think that would've been really difficult to place yourself on every day. (P9)*<br><br>*I think it was pretty easy to track pain because 1 to 10 of "no pain" to "worst pain ever" and having the middle point of 5 is "moderate pain", that's better than just a 1 to 10 on its own. (P10)*
(iii) Reminder to check-in a helpful feature to engage users<br>Having a reminder to complete the daily check in was also highlighted as an important component in keeping users engaged with the app on a daily basis. Several participants commented that without the reminder, they would have forgotten to use the app.	*I did also quite like the notification bar that came down, because I would probably have forgotten more times if I didn't have that (P6)*<br><br>*Really good that that's there because I definitely needed a reminder each day (P3)*<br><br>*…it was giving you a notification and that was good. You forget about the app and maybe you downloaded it, you just let it go, but at a certain time every day it tells you "time for your check in". (P7)*<br><br>*I liked how the app sent reminders for the daily check in, which I thought was quite handy. (P8)*

	The check in, I liked the notices …so with regards to check in I feel as if it's good. It helps remind me every six hours or something to, for lack of a better word, check in (P11)<br><br>*Yeah, I thought it was really helpful and the notifications setting was really good, to be able to have that reminder as well. (P12)*
(iv) Visual diagram of mapping pain on body and using words to describe pain is engaging and interactive feature.<br>For a few participants, when first creating their profile on the app, they found the interactive mapping of where they experienced pain on their body and selecting words to describe the pain at these sites was an innovative feature which immediately engaged them.	*…so when you sign in and you make your little profile account you get a picture of a body and you can record your pain areas. You can click on different parts of the body and once you select the parts of the body it has little segments, for example like the neck, back of the head, chest, arms. Then, once you've selected a group of them, you then can select the describing words about that pain, you can click a couple of them. I really liked that. I haven't seen anything done like that, but I thought that was such a valuable tool and you could save a couple of different pain areas (P2)*<br><br>*I really liked the points of pain on the map of the body; I thought that was very, very well done. (P10)*<br><br>*I felt as if labelling the areas at the very beginning, whilst it was time-consuming, it was something that really helped ease the - I guess it made it easy to report pain. (P11)*
Subtheme 3.3.2 Information interactivity features appealing<br>There were two main information interactivity features that were identified by participants as enhancing usability. These were:<br>(i) Having the app recommend information articles to user based on daily check-in or trends in pain monitoring.<br>(ii) Being able to connect with other users of the same age group with similar issues and relate to them.	
(i) Having the app recommend information articles to user based on daily check-in or trends in pain monitoring.<br>Having the app recommend information articles based on their pain monitoring information was perceived by participants as helpful in actively assisting them with their self-management. Tailoring of information to the specific needs of users was an appealing feature and motivated participants to engage with the app on a regular basis.	*it already recommends certain articles to you, which is nice… it will provide suggestions based on what you're saying in your check-ins each day, which I thought was pretty good too because I personally have a bit of trouble with sleep, so it was nice to see that it was suggesting certain ideas as far as goals that you could set for yourself for sleeping. (P1)*<br><br>*It asks you how much sleep or if you've had a decent night's sleep. For me, generally the answer to that is no, but they give you articles. If you've been tracking very badly with your sleep, they do provide some suggested articles on sleep and how you can better manage it. It is very helpful, I think, because you might not realise that you're maybe doing the wrong thing before you do go to bed. Like watching something on TV before you've gone to bed, obviously that's very stimulating and a lot of people*

	will do that because you just want to sit on the couch and do nothing if you're in pain. So it's finding other things to do instead to wind down before you go to sleep, things like having a hot shower or things like that; I think a lot of people don't realise that that's better to do than to sit there and watch something on TV. (P1)<br><br>*I think after you enter it for three days it starts giving you some recommendations and patterns, which was nice. It gives you feedback and I think it was giving some suggestions of articles, which was good, then you click on them and start reading. (P7)*<br><br>*I really liked how they had the different articles on how to be active when you have pain and what exercises are recommended for when you're having pain. I really enjoyed that because that can be quite difficult. (P9)*<br><br>*Yeah, I really liked how there were different suggestions on what you could do to improve your sleep. I found them really helpful, because I've been having a lot of trouble sleeping lately, to put them into my everyday life and improve my sleep. (P9)*<br>*I liked how if you put in for a couple of days that you'd had bad pain, I liked the suggestion of the articles. I found that really useful and then I think there was a button you could click to go back and find those articles so that you could save them if you didn't want to necessarily look at them then and they provided a lot of good information to be able to refer back to. (P12)*
(ii) Being able to connect with other users of the same age group with similar issues and relate to them.<br>Being able to connect with peers who had similar conditions and share experiences and information as well as having the opportunity to discuss issues or challenges they may be facing was another interactive feature of the app that was highly appealing to participants. One user highlighted that question prompts from the app also made it easy to start talking to other users and reduced uncertainty as to how to initiate a conversation. A few participants also noted that it made them feel less alone.	*I did like that they have a community section on there as well because I think a big thing is not really knowing who to talk to that has similar issues to yourself. It's very hard to find anywhere that's like a support group for pain management when you're younger because I feel like it's more of a, well, any support groups seem to be aimed at people that are more middle aged rather than in the younger generation. the fact that it prompts you with certain questions as well on the community section, so it'll ask things like "How do you normally deal with your pain?" or "What other activities do you find you enjoy doing?" like hobby-wise and everything. It's a nice incentive to start you off if you're not sure how to first start talking to people about it (P1)*<br><br>*I think it made me think that I'm not alone, I'm not the only one going through this, and being able to identify that, like sometimes I would get insomnia, and how to deal with it. (P6)*

	I thought it was really good to be able to interact with other people who have similar issues that you do. Yeah. It's so difficult because no-one else at my school really understands, so I sometimes feel like an outsider. (P9)<br><br>*but I really liked that because, especially when the app gets bigger, it's more well-known and that kind of thing, you can talk to other people your own age. I think that's really, really important because there are some Facebook Groups and that kind of thing, but our generation isn't really on Facebook, so it makes it really simple and easy. (P13)*
Subtheme 3.3.3 Copey, a divisive character – mixed perceptions regarding the acceptability of the app icon for the target user group<br>The app icon 'Copey' was divisive, with a majority of participants disliking the icon. Participants across age groups indicated that it was too childish for the target user group and more appropriate for a younger audience. A few participants suggested that a cartoon character may have broader appeal if users were able to choose from a range of characters based on personal preference. However for other participants, Copey was perceived as a likeable cartoon figure that made it lighthearted and fun using the app.	*Dislike*<br>*I guess the design was a little childish… It's* [Copey] *not really needed, but it could be anything really. (P4)*<br><br>*I quite liked it, but I think it might be a bit too childish for my demographic. The icon is iconic, but I think that's what is making it childish for the younger group. I don't think it will be too much of an issue, but if you were going to change something it would probably be the little guy. (P6)*<br><br>*The logo, that monster sort of thing, the blue thing, I wasn't sure what that was. It was unclear. Maybe if that monster had a name or something, "Hey, my name is this, I'm going to help you today" that might be good. I don't know if there was a name and I just don't remember it. Sometimes you ask, "What's this monster here?" Maybe linking that to something might be good, bringing a meaning out of that creature... I can assume that it would be maybe something very nice for children, but for young adults it might not be. It's just that I think that monster aspect made it a bit childish…If when you first put your age in that brings up a different icon or a different monster, depending on your criteria. I would think that putting a picture in the app depending on the age group is something very easy to be done (P7)*<br><br>*Once again, I think it was a bit too childish. Yeah, I don't know, it didn't really appeal to me. It was maybe something suitable for up to 15 year olds, but not 15 to 25. The app is based on a little monster person, so that stood out a lot. I think that was one of the things that I think is a bit too childish, that I didn't like as much But I don't know, I guess it gives it a bit of a character. … Yeah, it was a bit too much for*

	me and I think it was a bit overwhelming. It was like, "Ooh, I don't think this is for me" type thing (P8)<br><br>*I think it is. I think it's a little childish, but I think that young adults are returning to enjoying more childish things… maybe having the icons be a little bit more adult. (P9)*<br><br>*I hate the little cartoon. I think someone must've worked very, very hard on these little avatars, but I think they're hideous. I think that it is to a younger demographic, but the reason why I don't like this in particular is because there was a campaign led by Rail Safety in Victoria, it was called Dumb Ways to Die and the avatars look very, very similar. This is a Canadian app which means the crossover probably wouldn't happen, but my immediate association is, "Oh, this little blue blob is gonna die a hideous death right in front of me". (P10)*<br><br>*Yes, very much too juvenile. It was almost like "we're trying to categorise everyone who is sick as either being very young or very old" and it was quite alienating to me… Using the character was somewhat helpful, but I feel like maybe there should be either an edited character or some sort of, I don't know, something not as juvenile as the character used… Yeah, choose a mascot or something like that (P11)*<br><br>*I've downloaded one app that rivals this called GeoPain. I haven't been using this out of laziness because that's me, but I would attempt to use iCanCope, it's just the little monster gives me a deterrent against it. If it was released with suggestions and corrections etc. then I definitely would, but right now it's at a stage where it's not targeted towards my general demographic. It's very much obvious to users who are beyond the demographic it's targeted to…(P11)*<br><br>*The little character, some people could say that might be a little bit kiddish on the background, but I would say it would be fine. Because when you open the app it's quite a big feature, even just making it a smaller feature or a little logo or something, do you know what I mean? (P12)*<br><br>*It's a little bit of a grey area, I think. I think the initial home page with the monster was just a monster referring to - whilst yes, it allows them to access their inner*

	child, I think that's over the top for the target group. It's a little bit not quite with it. (P14)<br>*Like*<br>*Again, the cartoony side of things, it just really simplified what was needed in a very still effective way. and, again, using those cartoon characters was cool, I think. (P3)*<br>*I mentioned earlier I liked the little alien blob man. It kind of disconnects you a little bit from - I just think you have a little bit of a laugh as far as when it did have the little blob on the scoring scale it was a little bit cute of, you know, oh, they're feeling tired or they're feeling sick. I think yeah, that just can make people have a bit of a laugh at their situation, which I think is really nice. (P2)*<br><br>*That being said, I did like the graphics. I thought it was a cute animal and it was neutral, it wasn't gender-specific or really girly or masculine or anything. [P5]*<br><br>*and even the little people, like the little emoji thing or the monster was really helpful because it was really cute… Yeah, it was very colourful and the little monsters were really cute, so it made it more positive and really nice to look at. No, I think it hit the right age group because I think it's not too young, but it's nice, like it makes you laugh a little bit. (P13)*<br><br>*but it's meant to be fun and inviting. You can tell it's not for children, it's just something different to look at because obviously as well, maybe they've spent all day at work, they don't want to come home and be feeling like they're doing more work. Just sitting down, it looks like it's a game, it looks like it's just another app; it's not difficult work. Definitely it's fun. (P16)*
Subtheme 3.3.4 Multifaceted improvements for daily check in feature<br>Participants suggested a range of measures to enhance the usability of the daily check in feature, namely:<br>(i) As part of set up, allow the user to nominate the time for the daily check-in (default time midday).<br>(ii) Add a journal function as part of the daily check in to optimise self-management.<br>(iii) Allow for retrospective entering of information for daily check-in.<br>(iv) Allow users to be able to rate pain in different areas rather than just have one overall pain rating.<br>(v) Consider alternative scale for daily check-in that is more age appropriate [minor theme].<br>(vi) Additional notification/reminder features for daily check in [minor theme].<br>Major themes are discussed in detail below. Please note, minor themes are not discussed.	

(i) As part of set up, allow the user to nominate the time for the daily check-in (default time midday).<br>Participants highlighted that the default mode of daily check-in at 12pm was not intuitive given that most users were only part way through their day at that time and therefore were unable how to rate their pain, mood, sleep and physical activity for the entire day. Several participants had been able to work out how to change the default setting however others had not. It was suggested that to enhance usability, the user should be able to nominate their preferred time for the daily check-in when setting up their account for the first time.	*The only thing I could think of is the daily check-in thing…I think what actually would've been good is to have something pop-up at the start. I don't know if maybe I missed it and just skipped through it, but a pop-up at the start saying, "When do you want to be reminded each day to do your daily check-in?" Because I think I initially had it at 12 o'clock or something and missed it because I was at uni or something and it didn't come up as a notification at the right time of the day, so I saw it and then had opened my phone and the notification went away. Also, the fact that it was a 12 o'clock thing is like, well, that's not going to be able to summarise my day because then half a day still to go, I could do something in the afternoon that brings on my back pain. I mean, a very minor inconvenience, but then I had to go through the settings to find how to change my daily check-in time (P3)*<br><br>*I think when you're first setting up the account it would've been nice if it asked what time you want your check-in to be every day. I didn't actually realise until after the trial that you could change it in the settings. I should've looked, but I didn't. So it was defaulted to midday, which means I did actually forget on one of the days because I was at work and I just completely spaced on it being busy and then I missed it for the day. (P1)*<br><br>*you could put what time of day you wanted your check-in to be, because at first I kept getting them in the morning and it felt awkward 'cos I'd just woken up and they're like, "How has your day been?" I was like, "Well, I don't really know yet". So I switched that so that I got it about eight o'clock at night (P5)*<br><br>*I found that the notification that came up was about 12pm every day. I thought that was a weird time because it's asking about your entire day and if you'd woken up at eight o'clock, there was only four hours of information to go on. So I think pushing that back until 4pm or 5pm. Yeah. I think that I didn't know that I could change the check in time. That would be something to try and say when you're logging in, when you're setting the account, it could be, "What time would you like to check in?" (P6)*<br><br>*I found that it was a little difficult to set notifications for it because I was getting notifications in the middle of the day every day, but I couldn't work out how to*

	change it to a more appropriate time for me…. At the end of the day, around maybe 4.30. (P9)<br><br>*Yeah, it doesn't have anything that specifies [what time] I'm checking in and also it seems to let me check in multiple times a day. (P10)*<br><br>*another thing that I probably could've mentioned was maybe times of day when it's being recorded. Because I noticed at one time, for example, I wrote that I had zero pain and it was quite early in the morning and throughout the day, as the day went through, my pain actually got worse. And as well it was like, "Have you done any exercise today?" It was early in the morning, again, and I hadn't, but I ended up doing it later in the day… But that would be good, if it was a specific time of the day, like maybe 7pm at night or something, depending on the person, so it's recording the whole portion of the day, rather than a small portion of the day, so it can actually have a proper outcome and it'll be analysing over the whole period, rather than just maybe the morning or something like that. (P15)*
(ii) Add a journal function as part of the daily check in to optimise self-management.<br>Several participants wanted to be able to add notes or comments as part of their daily check in as a reminder of what activities or strategies they undertook during the day so they could identify any trends in terms of their self-management and ratings of pain, mood, physical activity and sleep. One participant highlighted that having such a journal function could also provide further opportunities for information interactivity where users could be prompted or reminded of logged self-management strategies that have worked in the past during their daily check in based on their rating scores.	*The next feature which I felt that it was missing out on was, again, like a step to be able to type in what advice have you received to reduce that type of pain to make people recall what advice they've had. Or when they do go to a physio or a doctor, they can type that into their app so that when they log into their daily check-in a similar pain in a similar area that they've experienced before it might pop up with "Have you thought about trying this? You've tried this previously and you've recorded this" … So I think there are some opportunities there because I felt this was a number scoring. I didn't really know what to do with that then once that was logged on the calendar and I got disinterested in logging my scores because I thought oh well, I'm not really sure what to do with this now… whereas I feel that it's got the basis that if you link that in with logging your self-management tools, I think that could definitely enhance the app and that would definitely be something that throughout my youth I would've loved using. (P2)*<br><br>*I think also having scales with your physical activity being the time limits that you did. So you know how sometimes you've got your 30 minutes or you didn't or you did, and if you did it would be good if you could enter the training that you did, like what physical activity you did. or even like a physical activity log I think could be good also where you could actually log, "Yes, today I did 30 minutes of walking (P8)*

	I feel like there should be, I don't know, multiple screens, in a weird way. So if you can swipe left or something like that and that can be your to do list or your notes and then swiping right could be something else, like a calendar, for instance. I guess that might just be the Millennial in me, but it feels like that [check in] *part is a little bit blank, to be honest. (P11)*<br><br>*but maybe if there was another other button to just add a little sidenote for that day, maybe like "I went to the doctors" and social occasions" or something like that, where you could add your own personal note to it. Yeah, like what was your pain level where you could put a 9 but then you could put "I've got an ear infection at the moment" or something, because it might not be the arthritis itself. So yeah, I think that would be helpful. (P13)*<br><br>*I think one thing was that whilst it's good for tracking, you can't really type out a little journal to yourself about what did you do on that day to help you understand or what did you do in order to get that pain to what level it is. I think they left that for self-management, to your own journaling and memory. (P14)*<br><br>*I think that would probably tie in better if there was a journal capacity, like you can write a few down or a few notes what happened on that day, what do you think caused your pain to be this bad or what was your - I think that would help young people more so to engage with the app a bit more so they can make a correlation, "Oh, I did too much exercise in one day and my back was sore". It will just allow them to track and monitor and then self-manage better, rather than going, "Oh, my back was sore yesterday, I did too much" because then you've got a constant history of it and you can reflect on it better than just pure anomaly. (P14)*
(iii) Allow for retrospective entering of information for daily check-in.<br>A few participants also wanted the app to allow for retrospective entering of information in the event that a user forgets to do their daily check in. This would allow users to maintain their pain tracking over time.	*I did miss one day in terms of the daily check-in and it would've been good to be able to, I mean, I couldn't figure it out and I'm not sure if you could or not, but to be able to go back one day to say, "Oh, for Saturday I actually felt like this, this and this. This is my mood", where it seemed like I could only do a check-in for the current day that I was in. So if you accidentally forgot one day, to be able to still be able to fill that in would be good I think. (P3)*<br><br>*I like using the app Clue and a feature that Clue has that I really like is you can retroactively post your stats. So if you forget to do it one day you can go back and*

	record how was I feeling yesterday, which is a feature that I really, really like and I'd like to see it in this app as well. (P10)
(iv) Daily check in modified so user able to rate pain in different areas rather than just overall.<br>Participants who experienced pain in more than one area of their body advocated for the pain rating in the daily check in to be changed from an overall level of pain to allowing the user to rate their pain in different areas of their body. This suggestion was perceived to enable the user to rate their pain more accurately, allow for more tailored pain tracking and also increase user interactivity.	*I felt though where it lost me a little bit is the regular check-ins were just a number scoring one to five. It did have a descriptor on it and it was a cute little - I liked the interface, it's a little blob alien, that was very cute, but I felt that where it was missing out on the opportunity is that each of those check-ins could have been checking in what areas of the body are in discomfort that day and what kind of pain that is. For someone who is just experiencing one type of pain long term that feature might not be beneficial, but I felt that having that ability to select the areas of the body and the descriptive words at each check-in rather than just a scale of one to five or one to ten would've been more helpful (P2)*<br><br>*The six or so, five or so questions, ask maybe having what kind of pain, like if it was an aching or a burning or a stabbing pain. You could possibly add whereabouts, if you wanted to go further into that. (P6)*<br><br>*For example, I put in two separate pain areas, so when it came to asking how was your pain today, I think it should be based on each section, it should be asking how your pain was in one section. For example, maybe one day I might not have had the same pain in the same area, the same level of pain, so I had to kind of rate it in-between both, if that makes sense, rather than maybe one day there was no pain somewhere and one there was really high pain.* (P15)<br><br>*Yeah, exactly. It's not realistic to the specific pain. I think it would be good if when you're rating your pain in all of those questions that you select which pain area you're referring to. (P15)*
Subtheme 3.3.5 Greater functionality (design features) to support end user self-management<br>Whilst there were several features in the app that supported end-user self-management (see Key theme 3.1), participants identified four main ways functionality could be improved to further optimise self-management:<br>(i) More support in stepping users through setting goals and integration with data and resources to assist in achieving goals.<br>(ii) More interactive questioning of users to provide more specific suggestions for self-management.<br>(iii) Easier access to print or email tracking history to treating health professionals.<br>(iv) Reminder function to revisit/monitor goals on regular basis.<br>These are discussed in further detail below.	

(i) More support in stepping users through setting goals and integration with data and resources to assist in achieving goals.<br>Participants wanted more assistance (in terms of the app prompting the user) to work through the steps they would take to achieve their set goal(s) or suggesting available tools to be able to assist them to monitor and progress their goals. Additionally, users indicated it would be helpful to be able to classify nominated goals into short-term and long-term timeframes and be able to set-up recurring or repeated goals to enhance usability.	*Yeah, kind of a similar message to what I've been saying through. I felt it was good to set the goals, but I feel like often if people aren't prompted to talk through how they're going to achieve that goal then that might not be as effective. But it depends how you read "goal" because it may be more like an action that people write down rather than a goal, like the action is there for effect rather than a broader goal. So I felt that needed a little bit more structure to it because otherwise I feel like people might, well, I felt that I was writing big goals, like, "Oh, this is the overall goal" but I was then looking for how do I achieve that goal, where do I put my actions in, but different people will probably interpret that differently. I feel like some talking through steps of thought processes of okay, well, you want to achieve this, well how are you going to achieve that, what tools have you got to achieve that already, what extra support are you going to seek to achieve that, just really stepping people through that thought process a little bit finer (P2)*<br><br>*Also with the My Goals, I think it would be important to link that to your normal check in every day. The information that you provide in your My Goals may be relevant to your check in in terms of exercise, sleep, or how you're feeling today (P7)*<br><br>*I used that for one day, but the goal I set, I don't know, it completed it automatically. I didn't really do it properly. I think the goal, because I set a goal which was to exercise 30 minutes every day, but I think you've got to be more specific and write a goal for one day. You've got to be, I think, more specific with that. So it would be good if rather than it asks "What is your goal?" it could be "What do you want to achieve today or another day?" just the wording of the question… If it gave you the option, say, like a goal and then within that goal you could set little sub-goals, like milestones, I think that could be a good way as well. (P8)*<br><br>*I think there should be some sort of connection between the goals and like if someone's on an iPhone, like the Health App or something like that, because a lot of goals are fitness-related and a lot of goals are reliant on travel distance, so I feel like that has room for integration. (P11)*<br><br>*I did have a comment to make about the goals. You can't setup repeated goals. For example, I put 100 squats a day as one of my goals, but then I didn't feel like I could*

	check it off every day, it was just like I just check it off after the first day and it disappears, then I have to make another one. It's funny, because I have arm pains going through and typing out, like if I were to make seven squat goals and then check them as the week went on, that might be a bit manual. So in the end I just did one and I didn't do 100 squats the next day because I'd already checked it off my list. (P5)<br><br>*Yeah, I had a bit of a range. I had short term, I had long term. It's nice to see your long term goals on there, but if you only have long term goals then I feel like you don't really get them completed. (P13)*
(ii) More interactive questioning of users to provide more specific suggestions for self-management.<br>Older participants expressed a preference for greater interactivity in terms of the app using the daily-check-in information to prompt users to think how they could improve their self-management. In particular it would be helpful if users were given suggestions or links to online tools or information that may assist.	*I felt that there could've been utilisation - and some of the mental health apps, like the Headspace apps, I think Smiling Minds as well, they have options where you can enter text, again, like prompting more that cognitive behavioural therapy, kind of questions like what has helped you in this situation before or what will you do to improve. I don't know the exact question, but I feel like some more of that interactive question answering, encouraging that self-management and self-improvement of current situations could be really well linked in with that scoring. (P2)*<br><br>*I think something like self-exercises, exercises that you can do at home, that might be useful in terms of managing your pain, like physio, but it's very specific to the injury that you have, so pre-known information would be very difficult. Maybe that's something that can be done as well, people put their condition, the sort of pain they're having and, depending on that pain, there might be some specific articles about exercises that they can do very easily for five minutes and maybe that can be in the self-assessment tool as well, "Here's your exercise today" sort of thing (P7)*
(iii) Easier access to print or email tracking history to health professional<br>While participants found the pain tracking history extremely useful, they indicated that it would also assist if there were easy steps within the app to be able to email or print out the pain tracking history so it could be used as part of clinical consultations with their treating health professionals.	*I tried making a pain diary but it was just so inefficient because I had no template as to how to lay it out, whereas for apps like this. I feel like another detail should be getting the data emailed, like being able to request your data being emailed to you or your clinician. That's something I find very, very important (P11)*<br><br>*I couldn't quite find how to get to the statistics in that context, I'm not sure whether you can access those… So, if you get it out in doctors' offices and that kind of thing, getting the data would probably be really helpful for doctors, 'cos I know*

	my one wants me to do this ten minute one a night and I'm like, "Yeah, whatever". Even stuff like that; getting that data would be probably amazing for them. (P13)
(iv) Reminder function to revisit/monitor goals on regular basis **[minor theme]**<br>For one participant, it was noted that a regular reminder to users to revisit their set goals on a regular basis would ensure users maintain momentum in terms of achieving their goals.	*I didn't go back and check it all the time. I think that's maybe something, if I had a notification that popped up and said, "When was the last time you checked your goals?" or something that could be good (P3)*<br><br>*But keep in mind sometimes people just get annoyed with all these notifications on their phone and sometimes apps can be just a little bit pushy almost, it's like, "Leave me alone". Then in terms of the goal side of things, maybe once every - oh, it depends how long you use it for, but maybe once every four or five days or once a week or something like that just to say, "When was the last time you checked your goals?" Just because sometimes you get notifications for things like, "Oh, we haven't seen you on this app for a while, come check us out!" and they do that every couple of days and that can get annoying." (P3)*
Subtheme 3.3.6 Provide different mode settings to enable tailoring the app 'look and feel' to each user<br>A few participants advocated for users to be able to personalise the look and feel of the app based on individual preferences to optimise engagement. One participant highlighted that having a professional design mode would also assist users if they needed to use the app during work.	*This would be a lot of work, but lots of apps, they basically have two modes,* [for example] *there's a mode with cute little animals and then there's a mode that's a little bit more professional. I use the little animal version, but sometimes at work I open it up and I'm like, "Mmmm, maybe I should change this to the more professional version". Even just so that it doesn't look like you're playing games at work, it's like, "Oh, I'm doing this check-in". [P5]*<br><br>*I think that people should be given modes for accessibility for reading, like different fonts etc. just because you never know what someone's condition is and them not being able to read something on a pain website or app might be another trigger to them feeling useless…[P11]*<br><br>*The only thing I would suggest would be a change of background or some personalised settings so people can really make it their own and keep it engaging (P12)*
Subtheme 3.3.7 Scales used to rate physical activity need to be revised - not considered intuitive **[minor theme]**<br>A couple of participants commented that the scales used to rate physical activity were not intuitive and suggested they be revised so that it reflected the quantity of activity undertaken.	*Again, the scoring of how good the physical activity was. I didn't feel that fits, personally, for a scale, especially for someone managing pain, like what classifies as good. Again, I felt like potentially different categories could've been more beneficial there linking into, again, those tools for management., so maybe like what physical activity did you find beneficial today or something like that and it can be a few different options rather than just being a scale from one to ten. I feel like mood and pain for some people, they're more familiar with scaling those two things on a*

	number scale, but I felt the physical activity didn't quite fit with that scoring method for me, personally. (P2)<br><br>*Physical activity levels being levelled from "great" to "the worst" doesn't really make a lot of sense. If it were in terms of minutes, that would, I think, be easier to navigate. (P10)*
**Theme 3.4 Navigation**<br>Overall, participants were positive about app navigation functionality, with features indicating strong usability including intuitive design, easy to use and readily findable content. In particular, participants liked how there were prompts during first time use outlining how to use the different functions of the daily check in. However, a few participants wanted to see more navigation instructions for other sections within the app (i.e. outside of daily check in).	
3.4.1 Easy to use and intuitive<br>Participants unequivocally reported that they were able to familiarise themselves with the layout and features of the app very quickly, found it intuitive to use and sections were well labelled.	*Yeah, I think it was pretty easy to navigate. Again, similarly to the website, just a couple of key bars on the bottom to say "check-in information", whatever, "goals", that kind of stuff I think was really good and you could go from there pretty easily I found, yeah. (P3)*<br><br>*The navigation around the app was, I felt, really intuitive and really easy (P2)*<br><br>*It seemed really easy to use. Obviously, they have all their tabs down at the bottom so you can go into your check-in or the community section or the articles and all that sort of stuff. It's very clearly labelled and it's easy to find everything that you're looking for (P1)*<br><br>*it was very easy to find what you were looking for 'cos the headings were labelled very clearly. It was very easy to see what you were doing and it was a nice layout. It clearly showed you where everything was and how to get to it. (P1)*<br><br>*I found navigation really easy. I had a little look through and it seemed fine to me. It took me like two minutes, I guess, to figure it out, but I just kind of clicked all the buttons and there was no point where I was just like, "Oh, I can't find this thing, where did it go?" Everything was right there [P5]*<br><br>*It was easy to find everything and it had the main things down the bottom, check in, goals, library, community. I think that really identifies what the app's about as well (P6)*

	Even the Welcome Home page, that was quite good as well, so where it has Check In, Goals, Library and Community, you can really see what you want to do at a glance. (P8)<br>*Yeah, it was so easy to navigate because you could really just click on and swipe which section you wanted to be on. Once again, everything just seemed to flow from one thing to the next. (P9)*<br><br>*It's pretty easy. Everything's on the bottom. You don't really have that much to distract you with, which I found very good and simplistic (P11)*<br><br>*The navigation around the app was good. I think the layout of the bars is fine, but your right panel, you've got your profile but also the history and I think the history should belong on the bottom toolbar so you can then refer to it if need be to see how you've been tracking this week. (P14)*
3.4.2 More guidance on how to utilise sections not related to the Daily check in<br>A few participants indicated that further guidance on how to use other sections within the app, particularly Social Activities (where users can discuss/communicate) and Library resources would be helpful to optimise utilisation of functions.	*I think it might be good if they had it so you could also start articles yourself, like a thread yourself, if you wanted to talk about something specific. But otherwise it is good that they do have the prompt there to start you off, because a lot of people have trouble starting to talk about their pain and how they manage it or if they're not managing it very well (P1)*<br><br>*No I didn't [use My Social Activities], I actually wasn't really sure how to use it (P4)*<br><br>*I guess because you were being directed at the beginning, it was showing you how to do things, it was kind of like, "Here's this, fill this out". That was fine, but more towards when I was doing it myself I didn't feel the need to use the other tabs that were there. I didn't quite familiarise myself with them much. I wasn't sure what they were. So I think the main ones obviously that I was using were doing the pain every day, but the other sections I wasn't too familiar with or understanding well. Yes* [more guidance on other sections]*, because I think the reason I stuck to the first one was because I was instructed how to use it. Obviously, you can go into places yourself and look at it, but it's just a lot easier if you know why it's there. So yeah, I think definitely. (P15)*<br><br>*But when you first open the app you go, well, okay, which one do I read first? There are a lot of things. I think the subjects and titles are very self-explanatory, you feel that you know what you want and you browse and you go okay, I should click on this*

	and read this. But for those who don't know what to do, maybe there could be some suggestions about, "Do you want to start with this one?" and then once you read that one, then maybe at the bottom it'll give you other articles that are relevant to that topic as well. At the bottom there could be suggestions like, "Okay, would you like to read this?" so that you follow up on a pattern. (P7)
**Theme 3.5 Perception of overall acceptability**<br>Overall, the app was acceptable to all participants and perceived as a valuable digital tool to promote self-management of pain, with 24-hour access via their smart phones enabling a flexible fit within their daily routines.	
3.5.1 App was considered a valuable resource for monitoring and managing pain<br>Participants perceived the app as an extremely helpful tool in being able to monitor pain over time, set goals to improve self-management and have the opportunity to connect with individuals who are of similar age and facing the same challenges in terms of managing their pain.	*I think inherently it has value in the check-ins and in the goals that would motivate me to use it. (P5)*<br><br>*Yeah, I think I would use it just because I know myself, I'm not actually very good at managing my pain at the moment and it is nice to have something there that you can find information on or talk to other people on for support (P1)*<br><br>*I definitely think I would use them. I think they're really helpful to have in your life because you really look at how you're going and set goals for yourself and find better ways to manage your pain, rather than just keep it all locked up inside (P9)*<br><br>*Yeah, I think the app I would probably use on a daily basis. The app I really, really liked. I don't know whether I'm still able to use it but yeah, if I'm still able to use it now then I'd love to. I think the app is just amazing. (P13)*
3.5.2 App readily available using phone<br>As noted by two younger participants, having the app available on their phone and being able to access it whenever needed was another feature that contributed to high acceptability.	*And as I said, it's so helpful to have an app on your phone where if someone asks you a question then you can just call up an article that you can use in that situation to help explain it better. I thought that was so nice and I wish I had that when I was younger… as I said, it would just be so nice because you always have an information source on your phone and you could just use it whenever you needed it. (P9)*<br><br>*it's always good to have a space where you know you can come back to that. So the saving settings and stuff were good because you're able to heart the thing and then it's saved so you can go into your library, and I thought that was a really great tool so that you can go back onto it. (P12)*

META theme 4: LEVERAGING UPTAKE OF DIGITAL TOOLS	
**Key theme 4.1: Market desirable features to user group**<br>Participants provided rich insights into how digital tools could be leveraged to extend reach into the community and drive uptake by young people with pain. Key themes included a strong focus on marketing how the digital tools could help potential users, highlighting key functionalities and including testimonials from ‘real users’.	
Subtheme 4.1.1: How the tools can help the user<br>Participants strongly advocated advertising messages which focused on how digital tools could help the user to encourage uptake across the target population. It was perceived that there needed to be a “selling point” to potential users to first peak their interest to consider downloading the app or accessing the website.	*if you chucked some stats up on there - obviously you may not know the effectiveness of just the app by itself, but let’s just say you did a randomised controlled trial of using the app versus not using the app and see that it’s decreased their pain, then that would be a way to market it in a sense of saying, “Well, we actually know that regularly using this app will help to decrease and manage your pain” or whatever. So it’s got to be some sort of selling point to convince people as to why they should be bothered and why it’s actually going to help them with hopefully minimal input from there. (P3) [APP]*<br><br>*I think the main purpose of the app is unclear initially. I think that making people realise why they need the app is important in the initial stage. If it wasn’t for this study, for example, I don’t imagine myself going and downloading this app because, as I mentioned at the beginning, why would an app help me? I can just go on their website and search this information. Value pitching is important in the beginning, like why they should use the app.(P7) [APP & WEBSITE]*<br><br>*I think it’s important to say how easy it is to use. I was sold on the app at all with how easy it was to use and find things and everything, and even just for people to have a place where they check in once a day or every second day, because that can make all the difference. If you only see a doctor every month, it can make all the difference to go, “This is what’s been happening and this is how good or bad it has been”, so I think to encourage people to look at the app and see how easy it is (P12) [APP]*<br><br>*I think curiosity drives young people. If you make it available and say that it’ll help you track your pain and so on, I think that might motivate them for people who want to track or monitor what they did. (P14) [APP]*

	Just like obviously a lot of teenagers have sleep problems and all of that kind of stuff and then they've started getting pain and stuff at uni and if you advertise it in a way like "If you don't know why you're having this pain, maybe you can try and monitor it and see if it has any outcomes so you can better yourself" type thing. (P15) [APP]
Subtheme 4.1.2: Testimonials from users Similarly, having testimonials from "real users" or social media influencers providing feedback on how the digital tools had helped them or changed their self-management was also considered by participants as an important marketing feature to optimise uptake.	*With testimonials it's very easy, you can just put a picture of a model and you can just call that person "Mark" and say this is a testimonial, but if there was a real link, maybe a social media link or real information about that person, then that will be a very credible testimonial and that will be relevant for the audience. Because they are getting self-aware, they're also getting aware of the information that is out there on the internet nowadays and that's right, marketing is going to be a challenge and testimonials, of course, will help. They will hopefully start using it and say, "Okay, maybe people are getting a lot of help from this app, maybe I should just check it out" (P7) [APP]* <br>*I think people do often read what other people have to say about it, so I think that definitely* [testimonials] *would make a difference (P12) [APP & WEBSITE]*
**Key theme 4.2: Multifaceted promotion/awareness approach** Participants outlined several different advertising approaches to promote uptake of digital tools across the target population. These included referral from a health professional, social media advertising, promotion through other relevant health websites (links), educational institutions and chronic disease organisations.	
Subtheme 4.2.1 Referral from a health professional which can be utilised both within and external to the clinical consultation. Recommendation from a health professional was one of the strongest themes to emerge. Participants perceived that endorsement from a health professional would increase the likelihood of young people accessing the tools, particularly if it was incorporated as part of self-management outside of the clinical consultation. Additionally, some participants also thought that advertising of the tools in clinical waiting rooms could also be beneficial. However, it should be noted that one participant was not in favour of health professional referral indicating that they would likely be sceptical or wary if this was recommended to them by a health professional.	*In terms of getting it marketable and getting people involved with it, I think definitely you'd be probably getting a higher proportion of people who are in more severe pain who have either just seen a health professional and maybe the health professional has said, "Oh, have you heard about this app? It's a good way to check-in with how you're going and stuff". I think that could be a good way in terms of getting the health professionals involved because I think somebody in severe pain would likely go and see somebody about it, rather than have their first thought to download an app. It would be more of an adjunct to the other more hands on, in-person therapies available, like almost a follow-up kind of thing, just like, "Okay, I've been your actual physio for today, but here's something that can just keep an eye on you and see how you're going"… That's not to say that it wouldn't be effective by itself, but I think that's just probably where you'd get the most marketability with it just because I guess people typically wouldn't think that an app by itself could help fix their pain (P3) [APP]*

	I think advertising this website and app through a range of health professionals, so the health professionals on the ground can be talking about these services and these interfaces with their clients directly. During the consultation, yes, and that could be a range of different primary and allied health professionals or just even services, like posters in medical centres and things like that so parents or youths just see it like, "Oh actually, I'll have a look at that". But I do think it's important that the health professionals are also aware and they're trying to encourage it as well… I think advertising it through health professionals will help catch those that are not necessarily, I guess, find that they would benefit from support and help them seek some support when they might not have been looking for it. (P2) [APP & WEBSITE]<br><br>*I found the actual website trial, the other websites and the app, just because I went into one of the hospitals to see a specialist, otherwise I wouldn't have actually known that it was something that was there either. (P1) [APP & WEBSITE]*<br><br>*Coming from health professionals, I'd rather hear that rather than like [from social media], yeah (P4) [APP & WEBSITE]*<br><br>*I think maybe getting health professionals in collaboration with this app is also important. When you go the doctor and you're getting diagnosed with something, you get a recommendation of this app which will help you keep track of it. (P7) [APP]*<br><br>*having that a doctors' surgeries or having doctors or other allied health, like physios or anything like that, refer it. (P8) [APP & WEBSITE]*<br><br>*I think advertising it in the doctors' waiting rooms, I think that could be good… If I saw an ad for it in my doctor's waiting room, I think I'd probably look into it and download it from there. (P9) [APP]*<br><br>*Well, chronic pain clinics and stuff. Because the places that they've got the research things out, it would be good to have them there where they're advertising the research because I've seen it around in a couple of the clinics that I go to. I think that's kind of where you'll get the most, in chronic pain clinics or rheumatologists. (P12) [APP & WEBSITE]*

	I think that as a part of pain, often you're used to being recommended the same things and having some of them work and a lot of them not work. But then as a part of kind of like I have to prove that I tried it, you just try absolutely everything so the next appointment you can be like, "I tried that following your recommendations and this is what I still am dealing with". So I do think I'd definitely follow all the recommendations to a tee and would absolutely download it if it was recommended to me. Yeah. (P5) [APP & WEBSITE]
Subtheme 4.2.2 Social media advertising<br>Advertising the digital tools on social media was another strong theme to emerge. Instagram and Facebook ads were nominated as the most effective way to promote the tools on social media. A few participants also indicated recommendation from a friend or through 'word of mouth' via a social media post could also influence uptake.	*But yeah, the online platforms as far as Facebook and other social media would help catch individuals that are already on that path. (P2)*<br><br>*a lot of younger people are using technology all the time, so you would have to have something that pops up showing on there this is something that you can use if you have chronic pain and need more information or support (P1)*<br><br>*You could advertise it on social media…Instagram [P4]*<br><br>*Where do I think of myself seeing it? Either an ad on Instagram - I don't use Twitter, some people use Twitter - or on Facebook or an email (P5)*<br><br>*Definitely social media, like Facebook ads because those are annoying, but you also have to sit though and watch them if you want to continue watching the videos. I think that would be a smart idea and then just other Facebook ads or Instagram ads. (P6)*<br><br>*I think social media could be a big way. Particularly through Facebook or Instagram, having ads that pop up I think could be a good way. (P8)*<br><br>*Yeah, I think maybe Instagram would be good. I think that probably, other than in a doctors' room, that would be the other way that I'd be exposed of it and really take note of it (P9)*<br><br>*I think that online advertising is the best way to go about it…[so that] the disabled chronic pain corners of the internet were aware of these, I think a lot of people would get a lot of good use out of them (P11)*

	The most important thing is more or less advertising. You can advertise all you want in pain clinics, for instance, in psychological practices, but in reality what hits my generation the most is targeted online advertising. This is not necessarily for me, because I'm trying to decrease my time, but for the majority of my friends, I have conversations where they can't stop looking at their phone. So the fact is their phone is a part of them and it is something that is attached to their body and therefore the best way to maximise this engagement is to advertise, even just ads, God, I don't know, getting other chronic pain resources to advertise the existence of these things, like Twitter accounts, especially Instagram ads. Instagram ads are very, very popular now. (P11)<br><br>*I think it would just be all about advertising and making it as accessible as possible for young people and seeing how they go, so advertising it where the demographic is to try and get the most people involved. (P12)*<br><br>*I think a lot of people at this age tend to use things that are advertised on social media or what they see others doing…I guess it's kind of like the fitness apps. A lot of people use fitness apps to track, so I just feel that this is something that people would use as well and it would need a lot of motivation, if that makes sense? I just think it would need advertising so it gets out there.*
Subtheme 4.2.3 Links through other websites<br>A couple of participants indicated that links to the app/website from other health related websites may also be beneficial in promoting uptake.	*But if it popped up on certain websites, like Beyond Blue or something like that as well, because I feel like a lot of them probably go onto those sorts of spaces to look for support... Yeah, because it makes it seem like it is more reliable and a better source of information if it is popping up on something like the government websites (P1)*
Subtheme 4.2.4 Promotion through educational institutions<br>Promoting pain digital tools at schools or Universities was another suggested way to directly engage the target population.	*I think you've got to put the information out there and I think one way could be through maybe schools, even if it is just like a pamphlet saying, "If you experience this pain, we've got these websites, we've got these apps, there are tools available". I guess the same could go at a university or education system, even if there was a pamphlet or something about the apps…(P8)*
Subtheme 2.5 Promotion through specific age relevant chronic disease organisations<br>Similarly, promoting digital tools through chronic disease organisations that provide support services to young people was also considered another effective way to directly engage the target population.	*In WA I know there's a thing called Camp Freedom, which is an arthritis camp with the JIA, but they talk about quite a lot of different apps, they hand out brochures and that kind of thing. So that is a really helpful way to get in as well, because all these kids are dealing with pain, so that would be a really, really good point of call to get exactly where you wanted it. (P13)*

Key theme 4.3: Increase longer term engagement with digital tools by increasing interactivity features with users

Older participants (24/25 years) emphasised that with the millions of apps and websites available to users on a daily basis, it is essential to continually update and increase interactivity features for users to increase the probability of long-term engagement. It was perceived that without continuous improvement and interactivity, the pain digital tools could be redundant to users rather quickly.

I think there's a lot of really valuable information on the website and in the app, but I think it's that linking to actions and helping to guide people through thought processes, rather than just being information or data tallying. Because it will help engage people for longer with the website and with the services and with the app if they feel like they're making process via the engagement with those online and app mediums…. I …I feel like they're trying to get help and how to get more support, how to make the change in the individual is more what people are going to be looking for on the website and the app, rather than just that information. I think once they've got that source of support, that's when they're more likely to seek the more scientific information about pain, in the experience of, yeah (P2)

I think there needs to be a target, an achievement where the app can continuously keep people in the loop. That is very important, otherwise if you check in for months that's going to put you off. If there are only articles then you'll start realising, "Okay, I've read all the articles. What am I going to do now? The app is useless for me" and then people might start deleting or not using it. So there needs to be continuous intermittent achievements that will keep people in the loop… With this app, if they want to achieve that and not become a secondary app then this needs to be very active and there needs to be some interaction to use it. I can think of discounts, achievements when you check in, achievements when you read articles…Nowadays, developers are trying very hard to get people to start using apps and they're giving a lot of incentives. I know some banking apps that are giving $10 credit when you first download the app and put your details there, so you get a free $10 and that's a very good motivation to increase. So managing it yourself in that app market is very difficult I think but, at the same time, this app is essentially a needs-based app and people will want to come to it not just because they're getting benefit out of it, but also because they need it. (P7)

"…maybe they could build a forum within the website where everybody shares their ideas and touches base on things that maybe others are wondering and professionals aren't able to answer, but maybe you can get it from another person. So maybe there could be topics that everybody can open, make a topic and people comment on it and stuff, they ask questions. It has a bit of a Facebook Group atmosphere to it and a bit of a community there, so that might be something useful. that would be very helpful because I think the relationships that most people are sharing can be on a personal basis, but when you're discussing with a professional, you're usually on your guard, you don't give out yourself 100%. So maybe that's a way to achieve that…or for example, there were pictures of young people and maybe the picture of the person who was telling the story…maybe if the person is happy to share their information on the website - and that could be asked, if they're happy to give their social media links - so people can follow them and maybe ask them directly. So, for example, maybe their Instagram account or any other site where others can follow them, because if they are there on the website people usually take them as a role model or leader and they want to reach out to them, especially if the story that they are telling is very relevant. So they might want to follow to see what they are doing, sort of thing. (P7)
